# The Accumulation of Toxic Elements (Pb, Hg, Cd, As, and Cu) in Red Swamp Crayfish (*Procambarus clarkii*) in Qianjiang and the Associated Risks to Human Health

**DOI:** 10.3390/toxics11070635

**Published:** 2023-07-22

**Authors:** Lang Zhang, Ziwei Song, Yuntao Zhou, Shan Zhong, Yali Yu, Ting Liu, Xiaoping Gao, Lekang Li, Chiping Kong, Xinna Wang, Li He, Jinhua Gan

**Affiliations:** 1 Yangtze River Fisheries Research Institute, Chinese Academy of Fishery Sciences, Wuhan 430223, China; zhanglang@yfi.ac.cn (L.Z.); songzw@whu.edu.cn (Z.S.); zhouyuntao@yfi.ac.cn (Y.Z.);; 2 Department of Genetics, Wuhan University, Wuhan 430071, China; 3 Jiujiang Institute of Agricultural Sciences, Jiujiang 332005, China; 4 Key Laboratory of Control of Quality and Safety for Aquatic Products, Ministry of Agriculture and Rural Affairs, Wuhan 430223, China

**Keywords:** *Procambarus clarkii*, Qianjiang, heavy metal, risk assessment, CR_mm_

## Abstract

**Simple Summary:**

Consumers have expressed concern regarding the food safety of aquatic products in light of escalating global environmental pollution. The potential accumulation of heavy metals in red swamp crayfish, an omnivorous aquatic species, may present a significant risk to human health. Hence, a comprehensive multi-point sampling was carried out in Qianjiang, the foremost export city in China, followed by a meticulous risk evaluation of the red swamp crayfish originating from Qianjiang. In the study, we found eight significant interaction relationships that may be utilized to construct predictive models for the spatial distribution of heavy metals in crayfish tissue. The findings indicate that the abdomen muscle of crayfish does not present any discernible risk to human well-being. However, a minute fraction of crayfish hepatopancreas samples exhibited excessive levels of arsenic, rendering it inadvisable for excessive consumption.

**Abstract:**

Due to rapidly expanding crayfish consumption worldwide, the food safety of red swamp crayfish (*Procambarus clarkii*) is of great concern. China is the largest consumer and producer of crayfish globally. As of yet, it is unknown whether the main crayfish production cities in China are within safe levels of toxic heavy metals and metalloids. For 16 consecutive years, Qianjiang city ranked first in China in processing export volumes of red swamp crayfish. This study presents a comprehensive analysis of the enrichment levels and associated health risks of the species in Qianjiang. In our research, samples of four crayfish tissues, including the head, hepatopancreas, gills, and muscles, were collected from 38 sampling sites distributed in Qianjiang to evaluate the concentration levels of five heavy metals (Pb, Hg, Cd, As, and Cu). The concentration levels of all five metals in muscle did not surpass the national standard. Furthermore, eight significant correlations have been found. For further in-depth assess risk of crayfish in Qianjiang, estimated daily intake (EDI), target hazard quotient (THQ), carcinogenic risk (CR), and estimated maximum allowable consumption rates (CR_mm_) were evaluated in the abdomen muscle and hepatopancreas. The THQ values for each metal were found to be less than 1, while the CR values were below 10^–6^. Additionally, the CR_mm_ for adults was determined to be 17.2 meals per month. These findings, based on the analysis of five metallic elements included in this study, suggest that the consumption of crayfish abdomen muscle in Qianjiang does not pose any significant health risks. However, it is noteworthy that certain regions exhibit elevated levels of arsenic in the hepatopancreas, surpassing the national standard, thereby rendering them unsuitable for excessive consumption. In general, the findings can be used to provide guidance for safe dietary practices in China.

## 1. Introduction

Freshwater crayfish production and consumption have significantly increased worldwide in recent years, according to FAO (2019) [1]. Red swamp crayfish, *Procambarus clarkii* (Girard 1852), is one of the most prolific species of cambarid freshwater crayfish, and it is highly desirable for consumers worldwide because it is delicious and nutrient-rich [2]. Although red swamp crayfish originated from North America and Mexico, it is currently found in 40 nations across four continents after a century of introduction, and may further expand to other regions [3]. Red swamp crayfish accounts for over 90% of the world’s freshwater crayfish supply in Europe [4]. It has been found in 16 European territories, mainly including Italy, Portugal, France, and Spain [5]. More than 90% of U.S. crayfish are processed in Louisiana [6]. Red swamp crayfish was introduced in China in 1929, and about 208.96 million tons have been produced and consumed as of 2020 [7,8].

Red swamp crayfish is an omnivorous and relatively long-living aquatic animal that can easily accumulate pollutants in water, including various pesticides, herbicides, and especially heavy metals [9,10].The need for further evaluation of the impact of heavy metals and other pollutants in red swamp crayfish on human health arises with the promotion of the “rice–shrimp” cultivation model [11,12]. Red swamp crayfish from rice–crayfish co-culture fields exhibit detectable levels of various heavy metals, such as manganese (Mn), mercury (Hg), barium (Ba), cadmium (Cd), lead (Pb), and chromium (Cr) [13,14]. A study on water quality monitoring conducted in southern Spain revealed the presence of the herbicide oxadiazon (10 ng/g) in crayfish, which was found to be linked to rice agriculture activities in the sampled region [15]. However, several studies have also documented the identification of heavy metals in *P. clarkii* when it is engaged in the non-crayfish–rice co-trophic mode [16,17,18]. According to prior research findings, the consumption of crayfish has been linked to various human ailments, including Haff disease [19,20], human paragonimiasis [21], and Vibrio infections [22,23]. These diseases can result in the manifestation of symptoms such as muscle weakness, gastroenteritis, wound infections, septicemia, rhabdomyolysis, fever, and cough. The presence of hemoptysis further complicates the diagnosis, as it may be mistaken for tuberculosis [19,20,21,22,23]. The ingestion of heavy metals can elicit comparable symptoms, such as nausea and cough, in human individuals. In more severe instances, it can precipitate the development of pulmonary emphysema and pulmonary fibrosis [24]. Numerous studies in the USA, China, and other countries have shown that the concentrations of heavy metals are increased in crayfish [13,25,26]. These findings suggest that the assessed concentrations of heavy metals in red swamp crayfish could also potentially pose a significant risk to human health.

High concentrations of essential trace elements, such as copper (Cu), are relatively toxic to humans [27]. However, non-essential metals and metalloids, such as cadmium (Cd), arsenic (As), mercury (Hg), and lead (Pb), are severely harmful to organisms, even in small concentrations [28]. About 100 mg of Cu in the human body can function as a structural and catalytic cofactor for various enzymes, including hydrolytic, electron transport, and oxygen utilization [29]. High Cu concentrations cause oxidative damage and cell death by facilitating Fenton-type redox reactions [30]. Ingestion of foods containing Cd may cause vomiting and diarrhea through the acute toxicity of Cd. Additionally, chronic exposure to cadmium may cause kidney damage, bone damage, and cancer [31]. Metalloids, such as arsenic, are ubiquitous on earth, and low doses and prolonged exposure to arsenic cause various medical consequences known as “arsenicosis” [32]. Acute As intoxication can cause organ damage and death. Furthermore, disfigurement of extremities caused by chronic As exposure may result in malignant tumor formation [33]. Pb is the second most dangerous metal after As. Increased Pb blood concentrations affect behavior, cognition, hearing, puberty, and the growth of infants and children. Meanwhile, Pb causes cardiovascular, neurological system, kidney, and reproductive issues among adults [34]. Hg exposure in humans and animals occurs mainly through fish intake [35]. Long-term exposure to Hg compounds in various forms (soil, water, food, and air) affects the skin, gastrointestinal, cardiovascular, urinary, nervous, and pulmonary systems [36,37]. Therefore, the threat to human health due to exposure to heavy metals in crayfish requires further analysis. Humans mostly eat red swamp crayfish for its abdomen muscle, while its other tissues are frequently used for flavoring [38]. 

Aquatic organisms frequently serve as reliable indicators for assessing the extent of environmental contamination [39]. A bioindicator encompasses a wide range of organisms, including microbes, plants, fungi, and animals [40]. In the context of aquatic animals, the assessment criteria may encompass dimensions such as the physical dimensions of the organisms, the concentration of toxins within their tissues, or the frequency at which deformities manifest within the population [41]. Fish are frequently utilized as a biological indicators to detect the presence of heavy metal pollutants within aquatic environments. The study conducted by Luczynska et al. employed perch, *Perca fluviatilis* (L.), and roach, *Rutilus rutilus* (L.), as indicators of heavy metal pollution in Lake Pluszne, located in northeastern Poland. Their findings indicated a higher concentration of mercury in the muscles of Perch and roach, while perch exhibited a greater propensity for enrichment of zinc and copper in comparison to roach, with the exception of zinc in muscles [42]. According to the research conducted by Nyeste et al., it was observed that the highest concentrations of trace elements were detected in juvenile organisms. This observation suggests that the trace element patterns found in the tissues of juveniles could serve as reliable indicators for assessing recent pollution levels in watercourses [43]. However, bivalve mollusks and crustaceans are widely regarded as the most preferred organisms for academic research on metal pollution due to their substantial abundance and rapid pollutant accumulation rates [40]. The use of crayfish as an indicator of environmental heavy metal contamination has been studied in Egypt, Spain, China, and many other countries [25,43,44,45,46]. An example of this is the development of a particular contamination index, known as the toxic contamination index (TCI), which was created to evaluate the degree of toxicity in polluted areas located in central Italy [44]. Therefore, it is necessary to determine the heavy metal levels in crayfish, which can not only protect human health, but also timely control environmental pollution.

China is the world’s leading crayfish producer. For example, FAO showed that crayfish production in 2017 (in China) exceeded 1 million tons, with a value of 37 billion USD, representing 80% of global production [38]. Hubei Province (central China) produces the highest amount of crayfish in China, accounting for 32.3% of the total crayfish production in 2021 [47] with an output value of 132.54 billion yuan in 2021 (http://www.hubei.gov.cn/hbfb/bmdt/202202/t20220221_4006309.shtml, accessed on 21 February 2022). Red swamp crayfish farming mainly occurs in Qianjiang city in Hubei. Moreover, many Chinese people buy crayfish from Qianjiang, indicating that the area is suitable for the analysis of heavy metals in crayfish and their associated health threats to humans. Although there are more wide-ranging studies on risk assessment, no study has reported on a single region teeming with crayfish [14,48]. 

In this study, a comprehensive selection of 38 distinct crawfish production bases in Qianjiang was undertaken, encompassing all geographical regions within the area. The objective of this study was to evaluate the distribution of heavy metals in crayfish, specifically focusing on the assessment of health risks associated with heavy metal contamination in Qianjiang crayfish. The analysis also encompassed variations in the bioaccumulation of five toxic elements, namely Pb, Hg, Cd, As, and Cu. Finally, a risk assessment (EDI, THQ, CR, CR_mm_) was conducted to derive informative recommendations for the management of food safety and environmental pollution.

## 2. Materials and Methods

### 2.1. Sample Collection

**Figure 1:**
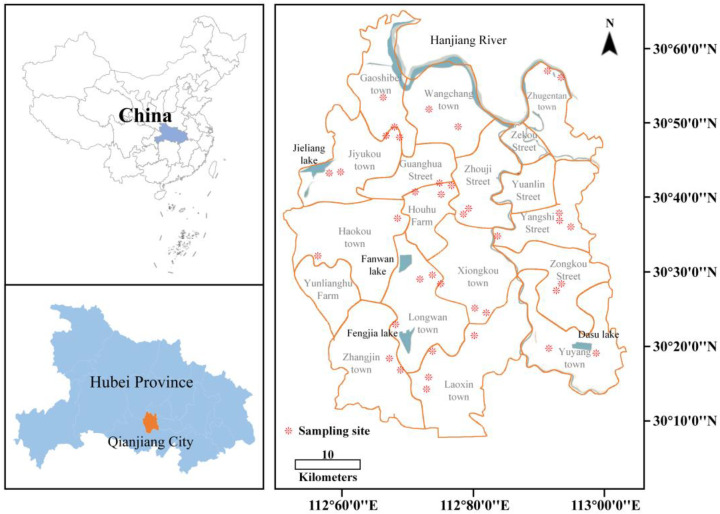
38 *P. clarkii* sampling sites in Qianjiang city, Hubei Province, China. Each * represents a sampling site.

The red swamp crayfish samples (18.43 ± 5.87 g) were collected from 38 sample sites across Qianjiang city (Figure 1), including 7 streets, 10 towns, and 6 management districts (from July to August 2019). A single crayfish farm of significant size was chosen for each sampling location, with three ponds being randomly selected from each farm for the purpose of sampling. Ten samples were collected from each pond, with three replicates (*n* = 10) obtained from three ponds for each sampling site. A comprehensive collection of 1140 crayfish samples was obtained from 38 distinct sampling sites.

### 2.2. Sample Treatment and Analysis

The samples were sealed in sterile polyethylene bags, packed into an ice box, labeled, then transported to the lab for processing. The crayfish samples were washed using distilled water. The live crayfish were weighed using an analytical balance (TG328A), and the hepatopancreas, head capsule, gills, and abdominal muscles of crayfish were resected from their whole bodies after anesthesia, and homogenized. Laboratory dissecting tools and containers were made of plastic or Teflon. To mitigate potential metal contamination, the dissection materials underwent an initial cleansing process utilizing a 20% (*v*/*v*) nitric acid solution, followed by multiple rinses with Milli-Q water. Nitric acid (65%, *v*/*v*, 7 mL) was used to digest each sample (0.2 g) in a MARS 5 microwave (CEM) under three steps at 120 °C for 6 min, 180 °C for 10 min, 190 °C for 15 min. The samples were initially cooled to the ambient temperature, following which the digestion tank was subsequently opened. Subsequently, the samples were subjected to heating at a temperature of 190 °C utilizing a graphite acid catcher (VB-40; CIL) until the volume was reduced to 1 mL. The samples were cooled to room temperature, then 25 mL of 2% (*v*/*v*) nitric acid was added and filtered through a 0.22 μm membrane. The same procedure was used to prepare the blanks (n = 10) and certified reference materials (CRMs, GSB-28, prawn constituent analysis standard material, n = 10) [49] for quality control.

Pb and Cd levels in the samples were detected using a graphite furnace atomic absorption spectroscopy system (AAS; AA240Z; Agilent, VARIAN, Palo Alto, USA). Cu levels were detected using flame AAS (AA240FS, Agilent). Hg and As levels were detected via fully automatic double-channel hydride generation AAS (AFS-3100, Agilent) (as detailed in Table S2) [50]. Blanks and duplicates were checked for every ten samples to ensure analytical precision. The CRM values of Pb, Hg, Cd, As, and Cu were 101.7 ± 3.8 μg kg^−1^, 79.4 ± 7.5 μg kg^−1^, 318.2 ± 45.9 μg kg^−1^, 236.5 ± 21.4 μg kg^−1^, and 2319.2 ± 195.3 μg kg^−1^ (dry weight), respectively. Standards for toxic components were spiked and digested to evaluate recovery. The limits of detection of Pb, Hg, Cd, As, and Cu (LOD, μg/g d.w.; back-calculated to tissue concentrations for each batch of analysis utilizing blank data) were 0.055, 0.084, 0.013, 0.091, and 0.042, respectively. The recovery result was within 10% of the verified values, demonstrating excellent precision and accuracy (as detailed in Table S3).

### 2.3. Health Risk Assessment

The maximum allowed levels (MLs) of metals in crustacea specified by Food Standards Australia and New Zealand (As: 2 mg kg^−1^ ; Pb 0.5 mg kg^−1^; Hg 0.5 mg kg^−1^; wet weight) [51], the Chinese National Food Safety Standard (GB 2762-2017) (As: 0.5 mg kg^−1^; Pb: 0.5 mg kg^−1^; Cd: 0.5 mg kg^−1^; Hg: 0.5 mg kg^−1^; wet weight), and the safe limit set by FAO/WHO (Pb: 0.2 mg kg^−1^; Cd 0.1 mg kg^−1^; dry weight) [52] were compared with heavy metal concentrations in crayfish. 

#### 2.3.1. EDI Calculation

Consumers may unintentionally consume some of the attached hepatopancreas when obtaining the abdominal muscle from cooked crayfish. Some consumers even prefer the hepatopancreas due to its nutritional value and flavor. As well as the hepatopancreas, the EDI value can be calculated using edible tissues of abdomen muscle, then compared with the threshold values of provisional tolerable daily intake (PTDI). The value of PTDI for As, Cd, and Pb is 0.83 × 10^−3^, 3.6 × 10^−3^, and 2.14 × 10^−3^, respectively [14]. The EDI model (Equation (1)) was utilized to calculate the accumulation risk value for five elements [50].

(1) $$\mathrm{EDI}=\frac{\mathrm{Ci}\times \mathrm{IR}}{\mathrm{BW}}$$

where Ci represents the hazardous element concentration in tissue (μg kg^−1^, wet weight) and IR represents the ingestion rate. The average daily intake per capita of crayfish in adult is about 10.54 g capita^−1^ day^−1^ (the intake of abdominal muscle and hepatopancreas is about 1.4229 g capita^−1^ day^−1^ (wet weight) and 0.6661 g capita^−1^ day^−1^ (wet weight), respectively) [14]. BW represents body weight (adult, 70 kg; and children, 20 kg) [26].

#### 2.3.2. THQ Calculation

THQ is widely used to assess non-carcinogenic human health concerns [53]. A THQ value higher than 1 indicates that human health is at risk [54]. Herein, the THQ value was determined as shown in Equation (2) [55]:

(2) $$\mathrm{THQ}=\frac{\mathrm{Ci}\times \mathrm{EF}\times \mathrm{ED}\times \mathrm{IR}}{\mathrm{BW}\times \mathrm{AT}\times \mathrm{RfD}}\times 10^{-3}$$

ED represents exposure duration (6 years for children and 78 years for adults). The risk from intermittent exposure was calculated as the sum of short-term exposure events based on the average approach in Equation (1), with EF equal to 120 d/year. This was performed because there is a significant seasonal variation in consumption, which leads to intermittent exposure. AT represents average exposure time (70 years × 365 days/year) [49] and RfD represents the oral heavy metal reference (safe) dose (μg kg^−1^ day^−1^; Pb, 20; Hg, 100; As, 0.3; Cd, 1; Cu, 40) [26,48].

#### 2.3.3. CR Calculation

A person may acquire cancer if exposed to hazardous materials, according to CSF (CR). A lifetime cancer risk of between 1 × 10^−6^ and 1 × 10^−4^ is acceptable based on USEPA (2000), while a lifetime cancer risk greater than 1 × 10^−4^ is unacceptable, and a risk less than 1 × 10^−6^ is negligible. Herein, CR was calculated using Equation (3) as follows:

(3) $$\mathrm{CR}=\frac{\mathrm{EF}\times \mathrm{ED}\times \mathrm{IR}\times \mathrm{Ci}\times \mathrm{CSF}}{\mathrm{BW}\times \mathrm{AT}}\times 10^{-3}$$

CSF represents USEPA’s Integrated Risk Information System (IRIS) online database’s oral carcinogenic slope factor (1.50 (mg/kg/d) for inorganic As and 0.0085 (mg/kg/d) for Pb) [56].

The MLs fish consumption rates for adults were calculated for both carcinogenic and non-carcinogenic effects to estimate the safest crayfish quantity that can be ingested over a certain period. The daily allowable maximum fish consumption for non-carcinogenic effects [55] was calculated as follows:

(4) $${\mathrm{CR}}_{\mathrm{limn}}=\frac{\mathrm{RfD}\times \mathrm{BW}}{\mathrm{Ci}}$$

(5) $${\mathrm{CR}}_{\mathrm{limc}}=\frac{\mathrm{RfD}\times \mathrm{BW}}{\mathrm{Ci}\times \mathrm{CSF}}$$

CR_limn_ and CR_limc_ represent the maximum permissible consumption rate (kg/day) of fish with non-carcinogenic effects and carcinogenic effects, respectively; the other parameters have been defined. About 3% of the total As element is considered to be inorganic. 

### 2.4. Estimated Monthly Crayfish Consumption per Person

Maximum safe monthly consumption rates (CR_mm_) [55] were calculated to determine the maximum amount of crayfish meals consumed each month for an entire year without causing a chronic consequence due to exposure of a specific metal I, as shown below:

(6) $${\mathrm{CR}}_{\mathrm{mm}}=\frac{{\mathrm{CR}}_{\lim}\times \mathrm{Tap}}{\mathrm{MS}}\;$$

CR_mm_ represents the maximum permitted intake of crayfish (meals/month); Tap represents average period (365.25 days/12 months = 30.44 days/month), and MS represents meal size (230 g/meals for adults, 82 g/meals for children; [55,57]).

### 2.5. Statistical Analysis

Data were statistically analyzed using SPSS (v24.0, SAS Institute, Cary NC, USA). Pearson’s correlation was used to analyze the association among variables (*p* < 0.05 indicated statistical significance). The normality of distribution for the variables was assessed using a Shapiro–Wilk test, which indicated a lack of normality (Shapiro–Wilk test, *p* < 0.05). The homogeneity of variances was examined through an ANOVA to investigate significant spatial and temporal variations among the five elements. Duncan’s test was used for multiple comparisons with two degrees of significance: *p* < 0.05 and *p* < 0.01. 

## 3. Results and Discussion

### 3.1. Concentrations of Toxic Elements in Crayfish Tissues

**Figure 2:**
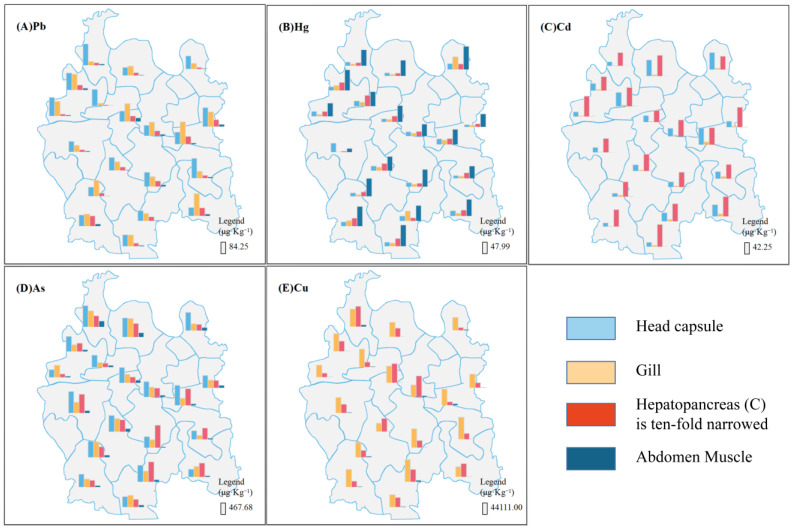
Accumulation of five heavy metals in different *P. clarkii* tissues in 38 sampling sites. The data are presented as the mean concentration of two adjacent sample points for Pb (**A**), Hg (**B**), Cd (**C**), As (**D**), and Cu (**E**).

The concentrations of the five toxic elements are shown in Supplementary Table S1 and Figure 2. The average concentration of heavy metals in the hepatopancreas and abdomen muscle are shown in Table 1. Mean conversion factors of 3.3 and 5.75 were applied to calculate metal levels in hepatopancreas [44] and abdominal muscle, respectively, for easier comparison between wet weight results and dry weight published data [26]. The concentrations of Pb, Hg, Cd, and As in abdominal muscle were below the Chinese national safety levels (GB2762-2017), World Health Organization WHO (1989) standards, USEPA (2000), and (FSANZ, 2013) [51,55]. Cu levels are not specified in national standards. The concentration of Hg and Pb did not exceed the national standard in other tissues (head capsule, hepatopancreas, and gills). Cd concentrations in most tissues did not exceed the national standard, except the Cd levels in the hepatopancreas. As levels in some sampling sites were higher in the head capsule (30), hepatopancreas (16), and gills (14) than the standard. Cu concentrations were relatively higher than those of other heavy metals in three tissues by more than ten times (Supplementary Table S1). As and Cu concentrations were significantly higher in the four tissues than the other three heavy metals. Notably, the Cd concentration was extremely low in abdominal muscle (Pb: 23.2 μg kg^−1^; Hg: 86.9 μg kg^−1^; Cd: 0.8 μg kg^−1^; As: 121.1 μg kg^−1^; Cu: 2782.1 μg kg^−1^). Metal concentration patterns in the hepatopancreas and abdominal muscle were as follows: Cu > Cd > As > Pb > Hg, and Cu >As > Hg > Pb > Cd, respectively (Table 1); this is consistent with the results of Tan et al. (2021) [14]. However, Peng, Nunes et al. (2016) [48] and Xiong et al. (2020) [58] reported different results, possibly because of different locations and ranges of research indicating heavy metal pollution levels in different regions [59].

The concentrations of the five heavy metals were consistent with the results of previous studies (Table 1) performed in Anfusi County, another city in Hubei [58]. The distribution of the same metal across different tissues is presented in Figure 2. Each histogram in Figure 2 is based on the average metal concentration of two adjacent sites. Metal concentrations were significantly different among different regions of Qianjiang. Pb and As concentrations were higher in the head than in the other tissues. Nonetheless, the As concentration was higher in the hepatopancreas than in the other tissues in some sample sites (Figure 2A,D). Hg and Cd levels were highest in the abdominal muscles and hepatopancreas, respectively, (Figure 2B,C). Cu levels were highest in the gills in most areas. However, Cu levels were higher in the hepatopancreas than in the gills in other areas (Figure 2E). Notably, the concentration of the metals (except Hg) was higher in the hepatopancreas than in other tissues, consistent with results of Rodriguez-Estival et al. (2019) [26]. Most studies have shown that the hepatopancreas of red swamp crayfish is the primary organ for storing trace metals [25,60]. The higher Hg levels in the abdomen muscle could be due to the high affinity for sulphur-containing cysteine in Hg proteins [61]. Compared with other tissues, muscles accumulate large Hg amounts [62].

**Table 1:** Mean and range of elemental concentrations in different tissues of crayfish from various study areas (mg/kg, dry weight).

Area	Tissue	Metal					Reference
		Pb	Hg	Cd	As	Cu	
China, Hubei, Qianjiang city	He	0.228 ± 0.13	0.096 ± 13.27	4.04 ± 1.9	2.046 ± 2.036	116.69 ± 140.59	Present study
	CV	0.57	0.46	0.47	1.00	1.20	
	Am	0.139 ± 0.06	0.5 ± 0.26	0.005 ± 0.008	0.7 ± 0.2	16.0 ± 12.0	
	CV	0.43	0.52	1.70	0.29	0.75	
China, Hubei and Hunan	He	0.46 ± 0.15		3.54 ± 0.78	3.51 ± 0.83		[14]
	Am	0.13 ± 0.04		4.59 ± 1.67	0.56 ± 0.12		
China	Am	<dl-1.01		0.003–1.71	1.012	7.2–157.6	[48]
China, Hubei	Am	0–0.05	0–0.06	0–0.12	0–1.27	11.11–21.87	[13]
	Wc	0–0.65	0–0.05	0.05–0.31	0.67–4.05	38.37–85.62	
USA, Louisiana	He	9.15–10.03		0.22–0.65	0.18–6.79	18.46–23.95	[18]
	Am	2.44–4.49		0.06	0.15–3.67	23.9–34.3	
USA, California	Am	0.19	1.10	0.03	0.68	44.6	[62]
Spain, Ebro River	Am	0.41–4.2	0.22–3.1			12–82.3	[45]
Italy, central Italy and Lake Trasimeno	He	0.49–39.23		1.96–73.4		3.6–1310.5	[44]
	Am	0.5–3.25		0.13–5.59		12.4–327.3	
Italy, south-western Sicily	He	0.74 ± 2.80		0.03 ± 0.04	3.76 ± 2.71	41.0 ± 38.5	[63]
	Am	0.18 ± 0.62		0.01 ± 0.00	1.79 ± 0.8	17.3 ± 9.4	
Italy, Po River Delta	He	<dl-1.11		<dl-1.26	2.58–4.48	94.2–686.5	[60]
	Am	<dl-2.43		<dl	0.52–0.8	30.5–65.3	
Safety standards (w.w.)		0.5	0.5	0.5	0.5		GB2762-2017

He, Hepatopancreas; Am, Abdominal muscle; Wc, Whole crayfish.

### 3.2. Correlation Analysis among Five Toxic Elements in Crayfish Four Tissues

**Figure 3:**
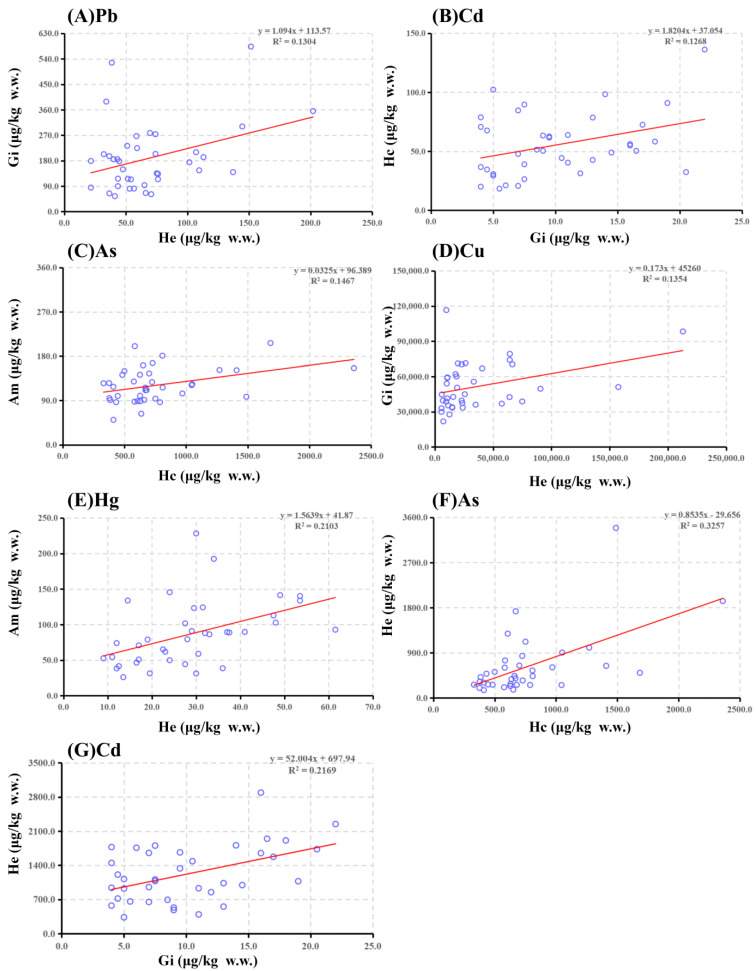
Linear regression analysis of the same metals in different tissues. The linear correlation was presented for Pb (**A**) and Cd (**B**), As (**C**), and Cu (**D**) between different tissues (*p* < 0.05), while Hg (**E**), As (**F**), and Cd (**G**) showed a strong correlation between the hepatopancreas and other tissues (*p* < 0.01). Hc, Head capsule; Gi, Gill; He, Hepatopancreas; Am, Abdominal muscle.

The correlation coefficients among different toxic elements in red swamp crayfish tissues are shown in Table 2. The heavy metal concentrations in the hepatopancreatic tissues were positively correlated with concentrations in other organs. For example, Hg levels in the hepatopancreas were significantly positively correlated (r = 0.459, *p* < 0.01) with Hg levels in abdominal muscle. Furthermore, Cd and As levels in the hepatopancreas were significantly positively correlated with Cd levels in the gills (r = 0.466, *p* < 0.01) and As levels in the head capsule (r = 0.571, *p* < 0.01), respectively. The concentration pattern of Hg, Cd, and As in the hepatopancreas may further suggest the role of immune organs in the hepatopancreas and the accumulation mechanism of different crayfish metals. The hepatopancreas is rich in both sulfide bonds and digestive enzymes, which are essential for the detoxification process. About 80% of the potential of hepatopancreas to accumulate heavy metals is due to sulfhydryl groups across lysosomes, which are facilitated by the presence of extremely complicated mucus [64]. Gills and intestines are the first organs in crustaceans exposed to external contamination, and are crucial for excretion and gas exchange [65]. Previous studies have shown that Hg accumulation in *P. clarkii* tissue organs is highest in muscular tissue [16,45,66]. Methylmercury binding through cysteine can reduce the bioaccessibility of methylmercury in muscle tissue [67]. The hepatopancreas generates oxidative stress under chronic exposure to Hg, affecting the synthesis of non-enzymatic antioxidants, such as tocopherols, ascorbic acid, and glutathione (GSH) [68]. Methylmercury can inhibit the rate-limiting enzyme of GSH synthesis, γ-glutamyl-cysteine synthetase [69]. Gills accumulate Cd faster than other tissues. Gills appear to be a transitory target for Cd accumulation when exposed to low Cd concentrations, leading to Cd transport to the hepatopancreas [70,71]. There are both inorganic and organic forms of As. Inorganic As is the most toxic type [72]. The International Agency for Research on Cancer (IARC) has categorized arsenic as carcinogenic to humans due to its association with skin, vascular, nervous system diseases, and cancer [33]. In addition, the Pb concentration in the hepatopancreas (r = 0.361, *p* < 0.05) and Cd levels in the head capsule (r = 0.383, *p* < 0.05) were significantly positively correlated with a corresponding metal concentration in gills, suggesting the mechanisms of heavy metal accumulation. Furthermore, a linear regression analysis was conducted on identical heavy metals present in various tissues (as performed in Figure 3), which can be employed to develop prognostic models for the geographical dispersion of heavy metals within crayfish tissue. However, this mechanism should be evaluated in more detail.

Multiple heavy metals have combined toxicity in vivo, showing additive effects, antagonistic effects, and synergistic effects [73]. Epidemiological evidence has suggested that combined Pb and Cd pollution is associated with higher incidence and mortality of cardiovascular disorders [74], demonstrating that Pb and Cd have synergistic harmful effects, which is consistent with the results of this study. Cd levels in gills were significantly correlated with Pb levels in gills (r = 0.359, *p* < 0.05). However, the nature of the combined toxicity may be either synergistic or contra-directional for the same toxin combination. Klinova et al. (2020) [75] discovered that Pb and Cd exert opposing effects on the blood pressure of rats. Therefore, many factors affect the type of interaction between metals [73]. In this study, As levels in gills were significantly correlated with Pb levels in crayfish gills (r = 0.616, *p* < 0.01). However, As concentrations were significantly inversely associated with Hg levels in the hepatopancreas (r = −0.400, *p* < 0.05) (Supplementary Table S7). The correlations of heavy metals in the same tissue may provide guidance for further investigation of the interaction mechanisms of metals.

### 3.3. Risk Assessment for Human Health

**Figure 4:**
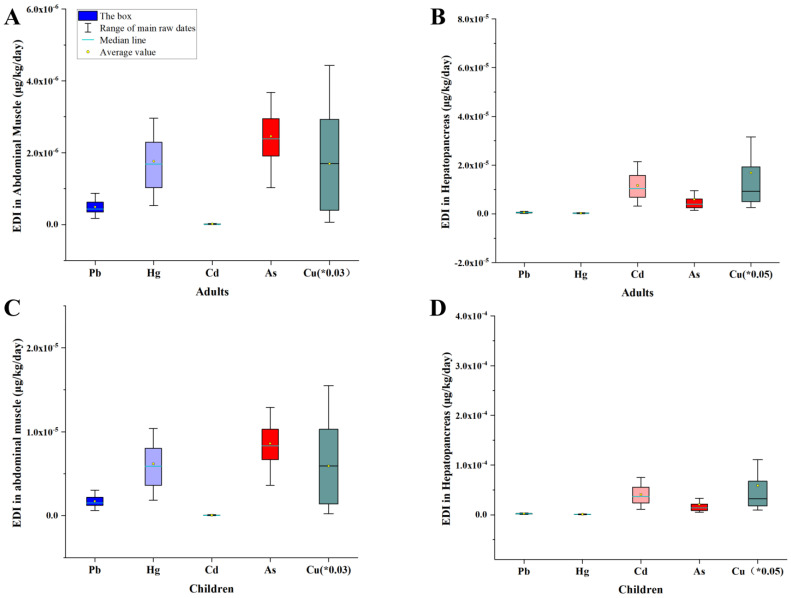
The EDI (μg/kg/day) of Pb, Hg, Cd, As, and Cu via the consumption of *P. clarkii* from Qianjiang. The EDIs of the abdominal muscle and hepatopancreas for adults and children are presented in (**A**–**D**), respectively. Due to the high EDI values of Cu, the displayed value is the original value multiplied by 0.03 or 0.05.

China is the leading crayfish producer and consumer worldwide, and more than a third of China’s crayfish comes from Hubei province [47]. In this study, the EDIs of the five toxic substances were calculated for children (20 kg) and adults (70 kg) based on toxic element levels in the hepatopancreas and abdomen (Mainland) [14]. The mean EDIs of Pb, Hg, Cd, As, and Cu for children and adults were all below 2.75 × 10^−4^ mg kg^−1^ day^−1^, which is significantly lower than the PTDIs [14] of toxic elements (Table 1). The EDIs of toxic components in both children and adults were lower than their PTDIs at the sites (Supplementary Tables S2 and S4). The distribution range of EDIs is shown in Figure 4. 

**Figure 5:**
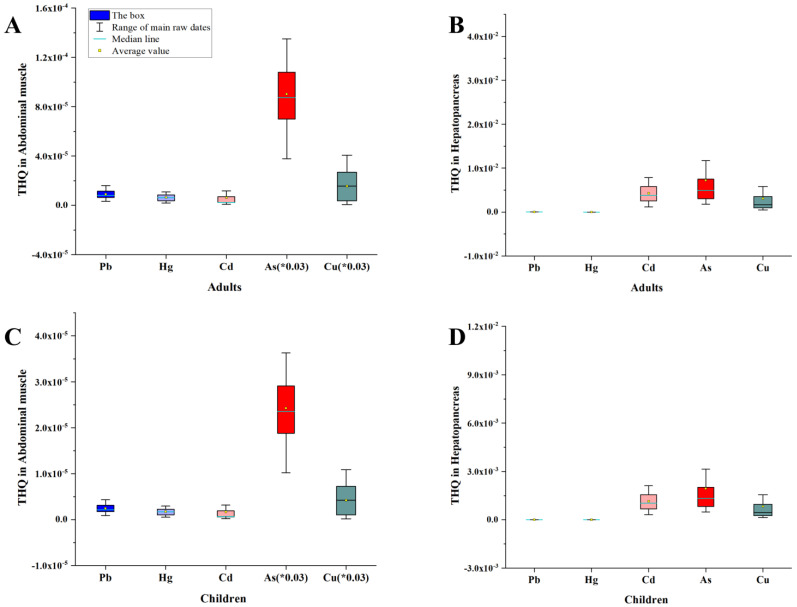
THQ values of Pb, Hg, Cd, As, and Cu estimated for children and adults via *P. clarkii* consumption from Qianjiang. The THQ of abdominal muscle and hepatopancreas for adults and children in (**A**–**D**). Due to the high THQ values of Cu and As, the displayed value is the original value multiplied by 0.03.

The mean THQ values for each toxic substance in the hepatopancreas and abdomen muscle are shown in Table 1. The THQ values for toxic compounds in the hepatopancreas and abdomen muscle tissues from all sites are shown in Supplementary Table S3 (children) and Table S5 (adults). The distribution range of THQ is shown in Figure 5. Additionally, As had the highest THQ value in the hepatopancreas in both children and adults, whereas Cd had the lowest THQ value in abdominal muscle (Table 1). Furthermore, THQ values for each toxin were less than 1 for both tissues (Table 1 and Supplementary Tables S3 and S5), indicating that the average daily intake of each of the metal was lower than the corresponding reference dosage in both children and adults. Therefore, a lifetime exposure to these concentrations of the metals may not have any harmful effects on humans.

Only CR values for As and Pb were measured, since only the CSF of As and Pb were provided (Table 3 and Supplementary Table S6). Higher As had higher CR values in the hepatopancreas than in abdominal muscle in both children and adults. CR values less than 10^−6^, greater than 10^−4^, and between 10^−6^ and 10^−4^ are deemed negligible, unacceptable, and acceptable, respectively [53,55]. In this investigation, the mean CR value for inorganic As and Pb in Qianjiang was less than 10^−6^, showing that ingestion of red swamp crawfish has no carcinogenic risk (Table 1). The CR values of As and Pb were less than 1 × 10^−6^ (Supplementary Table S6).

### 3.4. Safety Control Analysis of Crayfish Consumption

The CR_limn_ for all metals in red swamp crayfish was sufficient for human health protection (Table 3). The CR_limn_ for each metal (in kilograms) may not induce unfavorable non-carcinogenic health consequences [55]. As had the lowest CR_limn_ values, while Hg had the highest CR_limn_ values (Table 3). The results also showed that CR_mm_ can establish the maximum allowable limit of Qianjiang red swamp crayfish meals that can be consumed each month without causing any non-carcinogenic health problems. USEPA (2000) indicates that consumption is not advised where there is clear danger of exposure that could have adverse impacts on health (meals < 0.5/month), while consumption is safe and unrestricted where meals > 16/month. The CR_mm_ values of the five metals in muscle tissues were greater than 16 meals/month. The CR_mm_ values of Cd, As, and Cu in the hepatopancreas were greater than 0.5 meals/month and less than 16 meals/month, indicating that consumption of crayfish hepatopancreas from Qianjiang should be limited due to potential adverse effects on human health.

The CR_limc_ of inorganic As and Pb in abdominal muscle was 0.13 Kg/day and 3.4 Kg/day, respectively (Table 3). The CR_mm_ for As in adults was 17.2 meals per month (Table 3), indicating a safe consumption, since it is >16 meals/month [55]. USEPA (2000) proposes that lower CR_mm_ values for carcinogenic or non-carcinogenic consequences are crucial for human health protection [76]. The CR_mm_ for Pb was >180 meals/month (Table 3). These results indicate that the maximum allowable meals per month that individuals can safely consume for every metal considering noncancerous and cancerous health impacts endpoints is 17.2. The lowest CR_mm_ for children was 4.76, less than 16 meals/month, indicating that children’s consumption of crayfish should be controlled. The hepatopancreas contains significantly higher concentrations of toxic elements than the abdominal muscle (Table 1), which can lead to acute contamination with adverse health effects. Therefore, humans should not consume hepatopancreas.

## 4. Conclusions

This is the first study to comprehensively assess the risk of five heavy metals to human health in China’s most renowned crayfish producer, Qianjiang. The maximum allowable levels of heavy metals in abdominal muscle did not exceed the limits of international food standards. Hg levels in the hepatopancreas were positively correlated with Hg levels in the abdomen muscle (r = 0.459, *p* < 0.01). Also, Cd and As levels in the hepatopancreas were positively correlated with Cd levels in gills (r = 0.466, *p* < 0.01) and As levels in head capsule, respectively (r = 0.571, *p* < 0.01). Moreover, the EDI values of the five metals were significantly lower than the PTDI values. The THQ values for each metal were below 1, showing that the metals posed no health risks to humans. The CR for As and Pb was less than 10^−6^, showing that ingestion of Qianjiang crayfish carries no carcinogenic risk. The maximum permissible daily consumption rates were sufficient for protecting human health from non-carcinogenic and carcinogenic impacts. Adults and children can eat up to 17.2 and 4.76 crayfish meals per month with no harmful carcinogenic and non-carcinogenic health impacts, respectively, based on CR_mm_. However, consumption of hepatopancreas should be avoided since that specific geographical areas demonstrate heightened concentrations of arsenic in the hepatopancreas, exceeding the established national threshold. Moreover, the hepatopancreas has lower CR_mm_ values than the safety standards. These findings suggest that crayfish from Qianjaing is safe for consumption.

## Figures and Tables

**Table 2:** Correlation coefficients of the same toxic hazardous elements in different tissues of red swamp crayfish (*Procambarus clarkii*).

	Pb			Hg			Cd			As			Cu	
	Gi	He	Am	Gi	He	Am	Gi	He	Am	Gi	He	Am	He	Am
Hc	−0.167	−0.041	0.177	−0.073	0.178	−0.006	0.356 *	0.201	0.076	0.158	0.571 **	0.383 *		
Gi		0.361 *	0.291		−0.091	0.031		0.466 **	0.101		0.171	0.205	0.368 *	−0.073
He			0.305			0.459 **			0.193			0.008		0.119

Hc, Head capsule; Gi, Gill; He, Hepatopancreas; Am, Abdominal muscle; * *p* < 0.05, ** *p* < 0.01.

**Table 3:** Estimated oral reference dose (RfD, mg/kg/d) and cancer slope factor (CSF, (mg/kg/d)^−1^) for metals [57], daily intake for crayfish consuming populations (EDI, mg/kg/d), provisional tolerable daily intake (PTDI, mg kg^−1^), target hazard quotient (THQ), cancer health risk (CR), maximum allowable consumption rate of noncarcinogenic fish (CR_limn_, kg/day), maximum allowable consumption rate of carcinogenic fish (CR_limc_, kg/day), maximum allowable consumption rates in China (CR_mm_, meals/month).

		Tissue	Metal				
			Pb	Hg	Cd	As	Cu
RfD			0.02	0.1	0.001	3 × 10^−4^	0.04
CSF			8.5 × 10^−3^			1.5	
PTDI			3.6 × 10^−3^		0.83 × 10^−3^	2.14 × 10^−3^	
EDI	Adults	He	6.58 × 10^−7^	2.76 × 10^−7^	5.47 × 10^−6^	1.77 × 10^−7^	1.88 × 10^−4^
		Am	2.87 × 10^−7^	1.77 × 10^−6^	6.27 × 10^−9^	7.39 × 10^−8^	8.74 × 10^−5^
		He + Am	9.45 × 10^−7^	2.05 × 10^−6^	5.47 × 10^−6^	2.51 × 10^−7^	2.75 × 10^−4^
	Children	He	3.37 × 10^−6^	9.65 × 10^−7^	3.70 × 10^−5^	6.19 × 10^−7^	1.89 × 10^−4^
		Am	2.42 × 10^−6^	6.18 × 10^−6^	7.11 × 10^−9^	2.59 × 10^−7^	2.34 × 10^−5^
		He + Am	5.79 × 10^−6^	7.15 × 10^−6^	3.70 × 10^−5^	8.78 × 10^−7^	2.53 × 10^−4^
THQ	Adults	He	8.26 × 10^−6^	1.01 × 10^−6^	3.22 × 10^−3^	2.16 × 10^−4^	8.86 × 10^−4^
		Am	7.89 × 10^−6^	6.47 × 10^−6^	1.17 × 10^−5^	9.02 × 10^−5^	1.04 × 10^−3^
		He + Am	1.62 × 10^−5^	7.48 × 10^−6^	3.23 × 10^−3^	3.06 × 10^−4^	1.93 × 10^−3^
	Children	He	3.56 × 10^−6^	2.72 × 10^−7^	1.55 × 10^−3^	5.82 × 10^−5^	2.31 × 10^−4^
		Am	3.48 × 10^−6^	1.74 × 10^−6^	6.18 × 10^−7^	2.43 × 10^−5^	1.92 × 10^−5^
		He + Am	7.04 × 10^−6^	2.01 × 10^−7^	1.55 × 10^−3^	8.25 × 10^−5^	2.50 × 10^−4^
CR	Adults	He	2.05 × 10^−9^			9.73 × 10^−8^	
		Am	1.53 × 10^−9^			4.06 × 10^−8^	
		He + Am	3.58 × 10^−9^			1.38 × 10^−7^	
	Children	He	4.38 × 10^−9^			2.08 × 10^−7^	
		Am	4.13 × 10^−10^			1.09 × 10^−8^	
		He + Am	4.79 × 10^−9^			2.19 × 10^−7^	
CR_limn_ (Kg/day)	Adults	He	20.24	241.51	0.06	1.13	0.08
		Am	57.78	80.55	83.21	5.78	1.01
		He + Am	78.02	322.06	83.27	6.91	1.09
	Children	He	5.78	69	0.02	0.32	0.02
		Am	16.5	23.02	23.77	1.65	0.29
		He + Am	22.28	92.02	23.79	1.97	0.31
CR_mm_ (meals/month)	Adults	He	>180	>180	7.94	149.56	10.58
		Am	>180	>180	>180	>180	133.6
		He + Am	>180	>180	>180	>180	144.3
	Children	He	>180	>180	2.65	42.35	2.65
		Am	>180	>180	>180	>180	38.38
		He + Am	>180	>180	>180	>180	41.03
CR_limc_ (Kg/day)	Adults	He	1.19			0.03	
		Am	3.4			0.13	
		He + Am	4.59			0.16	
	Children	He	0.34			0.007	
		Am	0.97			0.036	
		He + Am	1.31			0.043	
CR_mm_ (meals/month)	Adults	He	157.49			3.97	
		Am	>180			17.2	
		He + Am	>180			21.17	
	Children	He	45			0.93	
		Am	128.38			4.76	
		He + Am	>180			5.69	

Hc, Head capsule; Gi, Gill; He, Hepatopancreas; Am, Abdominal muscle.

## Data Availability

Data are contained within the article and Supplementary Materials.

## Supplementary Materials

The following supporting information can be downloaded at: https://www.mdpi.com/article/10.3390/toxics11070635/s1, Table S1. Concentrations (µg kg^−1^, wet weight) of the five heavy metals in *P. clarkii* collected from Qianjiang, with detailed information of 38 sampling points. The data are presented as the mean ± SD. Table S2. The parameters of elemental determinations by GFAAS and AAS methods. Table S3. The values of the elements contents (μg kg^-1^) determind by given methods in CRM , recovery values (%), RSD (%) and LOQ values (μg kg^-1^). Table S4. EDI (mg kg^−1^ day^−1^) for children (20 kg). Table S5. EDI (mg kg^−1^ day^−1^) for adults (70 kg). Table S6. Target hazard quotient (THQ) of five toxic elements in children (20 kg). Table S7. Target hazard quotient (THQ) of five toxic elements in adults (70 kg). Table S8. Cancer risk (CR) estimate of As and Pb in children (20 kg) and adults (70 kg). Table S9. Correlation coefficients among four toxic elements in the same tissues collected from red swamp crayfish (*Procambarus clarkii*).
