# Supplementary material for: The Accumulation of Toxic Elements (Pb, Hg, Cd, As, and Cu) in Red Swamp Crayfish (Procambarus clarkii) in Qianjiang and the Associated Risks to Human Health
Source: Toxics. 2023 Jul 22;11(7):635. doi: 10.3390/toxics11070635 (PMC10384343; doi:10.3390/toxics11070635)
Supplement: Supplementary file 1 [file toxics-11-00635-s001.zip › toxics-2475532-supplementary.pdf]

Table S1. Concentrations (μg kg$^{-1}$, wet weight) of the five heavy metals in *P. clarkii* collected from Qianjiang with detailed information of 38 sampling points. The data are presented as the mean ± SD.

| Sample numbers | Farming area | Coordinate of sampling point | Total Pb content (μg/kg) | | | | Total Hg content (μg/kg) | | | | Total Cd content (μg/kg) | | | | Total As content (μg/kg) | | | | Total Cu content (μg/kg) | | |
|---|---|---|---|---|---|---|---|---|---|---|---|---|---|---|---|---|---|---|---|---|---|
| | | | Hc | Gi | He | Am | Hc | Gi | He | Am | Hc | Gi | He | Am | Hc | Gi | He | Am | Gi | He | Am |
| 1 | Yangshi Street | E112°94'38" N30°34'97" | 151.8 ± 16.7 | 203.2 ± 8.1 | 32.7 ± 3.6 | 14.1 ± 4.4 | 10.7 ± 1.2 | 7.0 ± 0.3 | 12.1 ± 0.7 | 38.5 ± 11.9 | 78.6 ± 6.3 | 13.0 ± 0.7 | 1035.0 ± 97.8 | 0.6 ± 0.1 | 1049.7 ± 376.3 | 520.7 ± 101.4 | 903.2 ± 203.5 | 123.2 ± 45.1 | 38720.0 ± 2578.0 | 9755.0 ± 892.1 | 4852.6 ± 974.6 |
| 2 | Yangshi Street | E112°83'42" N30°34'11" | 53.6 ± 5.9 | 174.5 ± 19.2 | 101.2 ± 11.1 | 11.6 ± 0.7 | 13.6 ± 1.5 | 11.5 ± 1.3 | 13.1 ± 1.4 | 26.1 ± 1.6 | 63.8 ± 8.3 | 11.0 ± 3.6 | 395.0 ± 31.7 | 0.3 ± 0.1 | 2360.3 ± 819.2 | 694.3 ± 274.3 | 1932.4 ± 651.7 | 155.7 ± 34.3 | 29900.0 ± 3994.0 | 5635.0 ± 639.4 | 3104.8 ± 467.2 |
| 3 | Yangshi Street | E112°91'88" N30°37'35" | 144.1 ± 5.8 | 116.6 ± 4.7 | 43.8 ± 4.8 | 34.0 ± 10.5 | 22.8 ± 0.9 | 13.2 ± 0.5 | 29.3 ± 3.2 | 123.3 ± 38.2 | 50.5 ± 4.7 | 9.0 ± 2.5 | 485.0 ± 49.5 | 0.1 ± 0.0 | 651.2 ± 197.1 | 398.6 ± 132.7 | 163.93 ± 21.4 | 161.39 ± 33.9 | 45000.0 ± 5877.9 | 25695.0 ± 3891.3 | 4915.1 ± 879.7 |
| 4 | Yangshi Street | E112°91'32" N30°36'89" | 178.3 ± 19.6 | 133.4 ± 14.7 | 76.6 ± 8.4 | 10.2 ± 0.4 | 20.4 ± 2.2 | 17.4 ± 1.9 | 27.5 ± 3.0 | 101.7 ± 4.1 | 56.1 ± 8.9 | 16.0 ± 5.2 | 2890.0 ± 297.8 | 0.3 ± 0.2 | 432.1 ± 129.4 | 292.5 ±73.2 | 482.1 ± 171.1 | 86.7 ± 33.2 | 71180.0 ± 6458.5 | 19730.0 ± 1845.0 | 1592.7 ± 471.1 |
| 5 | Zhugentan town | E112°93'23" N30°55'32" | 303.9 ± 33.4 | 85.9 ± 23.5 | 21.6 ± 2.4 | 23.2 ± 2.6 | 14.1 ± 1.6 | 23.8 ± 6.5 | 11.2 ± 1.2 | 54.3 ± 6.0 | 78.8 ± 9.1 | 4.0 ± 0.9 | 575.0 ± 86.8 | 0.3 ± 0.1 | 482.7 ± 193.3 | 275.1 ± 97.1 | 262.3 ± 74.6 | 142.2 ± 56.3 | 21830.0 ± 1896.7 | 7260.0 ± 797.6 | 4298.8 ± 598.2 |
| 6 | Zhugentan town | E112°91'22" N30°56'56" | 237.5 ± 26.1 | 149.0 ± 40.7 | 47.4 ± 5.2 | 17.6 ± 1.1 | 11.5 ± 1.3 | 29.4 ± 8.0 | 12.8 ± 1.4 | 41.6 ± 2.5 | 63.4 ± 11.4 | 9.0 ± 2.3 | 535.0 ± 102.1 | 0.3 ± 0.0 | 1044.6 ± 351.4 | 310.5 ±124.2 | 256.5 ± 68.3 | 121.4 ± 44.7 | 33010.0 ± 4988.3 | 5685.0 ± 673.4 | 910.8 ± 101.9 |
| 7 | Wangchang town | E112°77'23" N30°48'76" | 168.7 ± 18.6 | 267.1 ± 10.7 | 58.1 ± 6.4 | 21.2 ± 2.3 | 32.9 ± 3.6 | 15.6 ± 0.6 | 24.7 ± 2.7 | 145.5 ± 16 | 89.6 ± 15.6 | 7.5 ± 1.7 | 1110.0 ± 302.7 | 0.9 ± 0.4 | 445.3 ± 143.7 | 394.7 ± 87.2 | 268.1 ± 94.2 | 99.4 ± 27.8 | 70380.0 ± 10957.8 | 66500.0 ± 9785.4 | 328.7 ± 89.5 |
| 8 | Wangchang town | E112°72'89" N30°51'24" | 186.6 ± 20.5 | 135.7 ± 50.2 | 75.8 ± 8.3 | 25.3 ± 6.9 | 28.1 ± 3.1 | 18.7 ± 2.1 | 31.5 ± 3.5 | 124.2 ± 33.9 | 47.8 ± 7.5 | 7.0 ± 1.6 | 650.0 ± 79.2 | 0.1 ± 0.0 | 377.3 ± 109.5 | 392.1 ± 97.7 | 323.4 ± 139.2 | 95.0 ± 31.4 | 54040.0 ±1890.6 | 10165.0 ± 958.7 | 662.9 ± 123.5 |
| 9 | Guanghua Street | E112°70'77" N30°40'19" | 715.2 ± 376.7 | 61.7 ± 6.8 | 70.0 ± 7.7 | 34.7 ± 12.8 | 28.3 ± 8.8 | 13.5 ± 1.5 | 61.6 ± 6.8 | 92.9 ± 10.2 | 102.3 ± 19.8 | 5.0 ± 1.1 | 925.0 ± 157.9 | 1.0 ± 0.3 | 1683.8 ± 689.7 | 313.7 ±103.9 | 500.1 ± 213. 6 | 206.6 ± 81.7 | 36020.0 ± 2896.3 | 34950.0 ± 4588.9 | 5570.8 ± 784.3 |
| 10 | Guanghua Street | E112°74'69" N30°41'38" | 181.6 ± 20.0 | 179.4 ± 19.7 | 21.5 ± 1.3 | 20.4 ± 7.3 | 17.8 ± 2.0 | 21.4 ± 2.4 | 30.1 ± 1.8 | 31.3 ± 11.3 | 136.3 ±21.7 | 22.0 ± 3.7 | 2245.0 ± 474.5 | 1.6 ± 0.7 | 375.0 ± 38.4 | 480.2 ± 57.9 | 198.0 ± 27.8 | 125.5 ± 28.2 | 116570.0 ± 17765.0 | 9825.0 ± 791.2 | 1419.0 ± 497.2 |
| 11 | Jiyukou town | E112°66'54" N30°47'18" | 263.9 ± 29.0 | 178.4 ± 19.6 | 44.0 ± 4.8 | 21.5 ± 5.9 | 19.4 ± 2.1 | 21.8 ± 2.4 | 37.3 ± 4.1 | 88.9 ± 24.3 | 54.9 ± 12.4 | 16.0 ± 3.5 | 1650.0 ± 378.3 | 0.3 ±0.1 | 628.7 ± 89.1 | 376.1 ± 69.3 | 279.6 ± 78.1 | 88.7 ± 12.3 | 79220.0 ± 8940.1 | 64305.0 ± 4798.6 | 383.4 ± 57.7 |
| 12 | Jiyukou town | E112°68'67" N30°47'03" | 24.2 ± 2.7 | 90.5 ± 33.5 | 43.4 ± 4.8 | 12.7 ± 1.4 | 15.3 ± 1.7 | 22.2 ± 2.4 | 33.0 ± 3.6 | 86.4 ± 9.5 | 67.7 ± 23.2 | 4.5 ± 0.9 | 720.0 ± 101.4 | 0.3 ± 0.1 | 660.8 ± 101.2 | 217.9 ± 45.8 | 438.4 ± 56.2 | 91.2 ± 11.3 | 70420.0 ± 2044.0 | 23000.0 ± 3941.0 | 4149.2 ± 982.8 |
| 13 | Jiyukou town | E112°59'63" N30°42'58" | 387.3 ± 42.6 | 232.8 ± 9.3 | 51.7 ± 5.7 | 23.3 ± 2.6 | 66.2 ± 7.3 | 24.1 ± 1.0 | 53.2 ± 5.9 | 140.2 ± 15.4 | 61.6 ± 11.8 | 9.5 ± 1.7 | 1665.0 ± 297.4 | 3.8 ± 1.1 | 626.6 ± 91.3 | 515.6 ± 52.4 | 239.8 ± 63.4 | 99.8 ± 14.7 | 42730.0 ± 5789.0 | 15725.0 ± 2988.5 | 506.0 ± 89.7 |
| 14 | Jiyukou town | E112°57'99" N30°42'13" | 393.6 ± 43.3 | 389.7 ± 106.4 | 34.3 ± 3.8 | 20.4 ± 6.3 | 18.6 ± 2.0 | 13.8 ± 3.8 | 32.0 ± 3.5 | 88.0 ± 27.3 | 21.2 ± 5.4 | 6.0 ± 2.2 | 1760.0 ± 345.1 | 0.3 ± 0.1 | 727.8 ± 122.4 | 1553.1 ± 398.1 | 347.1 ± 72.7 | 166.0 ± 38.2 | 60080.0 ± 7080.0 | 18580.0 ± 3987.0 | 550.6 ± 71.4 |
| 15 | Gaoshibei town | E112°65'92" N30°52'65" | 567.7 ± 210.1 | 81.3 ± 8.9 | 53.8 ± 5.9 | 42.1 ± 4.6 | 12.7 ± 0.5 | 12.5 ± 1.4 | 27.3 ± 3.0 | 44.3 ± 4.9 | 34.5 ± 9.5 | 4.5 ± 1.2 | 1210.0 ± 179.3 | 0.6 ±0.2 | 575.9 ± 39.9 | 398.6 ± 41.7 | 211.5 ± 19.8 | 128.7 ± 29.1 | 38830.0 ± 4711.0 | 74945.0 ±7835.2 | 4807.0 ± 398.6 |
| 16 | Gaoshibei town | E112°67'63" N30°48'78" | 338.2 ± 121.8 | 81.4 ± 9.0 | 56.4 ± 6.2 | 17.3 ± 4.7 | 53.8 ± 19.4 | 23.4 ± 2.6 | 30.2 ± 1.8 | 228.2 ± 62.3 | 39.0 ± 7.9 | 7.5 ± 2.7 | 1080.0 ± 165.2 | 0.3 ± 0.1 | 330.0 ± 47.9 | 289.3 ± 27.2 | 262.9 ± 31.4 | 125.1 ± 13.4 | 35400.0 ± 3850.0 | 11380.0 ± 3520.0 | 3773.8 ± 981.3 |
| 17 | Zongkou Street | E112°92'42" N30°26'34" | 528.0 ± 58.1 | 66.4 ± 4.0 | 66.7 ± 7.3 | 31.0 ± 11.2 | 10.0 ± 1.1 | 8.5 ± 0.5 | 16.5 ± 1.8 | 46.6 ± 16.8 | 18.3 ± 5.8 | 5.5 ± 1.3 | 660.0 ± 97.3 | 0.6 ± 0.1 | 750.5 ± 78.6 | 288.6 ± 39.5 | 1120.5 ± 106.4 | 93.9 ± 19.7 | 33500.0 ± 3660.0 | 14935.0 ± 2673.5 | 517.9 ± 77.4 |
| 18 | Zongkou Street | E112°93'06" N30°27'34" | 300.5 ± 33.1 | 224.4 ± 69.6 | 59.3 ± 6.5 | 30.1 ± 3.3 | 13.9 ± 1.5 | 12.1 ± 3.8 | 30.7 ± 3.4 | 58.9 ± 6.5 | 42.7 ± 11.4 | 13.0 ± 2.7 | 555.0 ± 101.4 | 0.3 ± 0.0 | 723.8 ± 94.5 | 392.8 ± 71.0 | 836.4 ± 123.3 | 127.5 ± 12.3 | 58880.0 ± 4782.0 | 10615.0 ± 2891.4 | 587.6 ± 101.3 |
| 19 | Yuyang town | E112°91'18" N30°19'07" | 89.9 ± 24.5 | 583.5 ± 64.2 | 151.8 ± 6.1 | 59.1 ± 21.9 | 20.1 ± 5.5 | 14.7 ± 1.6 | 28.4 ± 1.1 | 79.4 ± 8.7 | 49.0 ± 9.2 | 14.5 ± 4.7 | 995.0 ± 178.1 | 0.6 ± 0.3 | 700.1 ± 149.7 | 1089.0 ± 137.5 | 645.4 ± 92.8 | 145.0 ± 23.7 | 49630.0 ± 4736.0 | 90545.0 ± 3678.5 | 4677.3 ± 578.1 |
| 20 | Yuyang town | E112°98'21" N30°18'14" | 272.4 ± 30.0 | 355.1 ± 39.1 | 202.7 ± 22.3 | 30.4 ± 11.0 | 26.9 ± 3.0 | 12.3 ± 1.4 | 23.2 ± 2.6 | 61.8 ± 22.2 | 51.4 ± 8.7 | 8.5 ± 1.6 | 695.0 ± 123.5 | 0.6 ± 0.2 | 670.7 ± 165.4 | 738.6 ± 94.8 | 1727.4 ± 249.8 | 111.0 ±23.2 | 37450.0 ± 3840.0 | 23530.0 ± 1487.0 | 310.6 ± 47.6 |
| 21 | Laoxin town | E112°72'42" N30°13'45" | 88.8 ± 3.6 | 278.1 ± 30.6 | 69.4 ± 25.7 | 30.8 ± 8.4 | 21.9 ± 0.9 | 14.9 ± 1.6 | 29.7 ± 3.3 | 91.0 ± 24.8 | 26.4 ± 9.3 | 7.5 ± 1.9 | 1805.0 ± 571.4 | 0.1 ± 0.0 | 410.6 ± 89.2 | 464.8 ± 45.5 | 154.9 ± 23.2 | 50.8 ± 9.7 | 62110.0 ± 6460.0 | 17698.1 ± 3144.2 | 2792.3 ± 598.4 |
| 22 | Laoxin town | E112°72'86" N30°14'75" | 399.2 ± 43.9 | 204.9 ± 8.2 | 74.6 ± 23.1 | 23.3 ± 1.4 | 14.5 ± 1.6 | 12.6 ± 0.5 | 37.0 ± 11.5 | 89.4 ± 5.4 | 50.4 ± 12.9 | 16.5 ± 5.5 | 1945.0 ± 643.2 | 1.0 ± 0.4 | 499.5 ± 48.3 | 528.4 ± 63.1 | 516.9 ± 79.4 | 149.9 ± 34.4 | 42540.0 ± 5280.0 | 64055.0 ± 7834.0 | 5293.0 ± 739.1 |
| 23 | Laoxin town | E112°73'29" N30°18'43" | 222.1 ± 24.4 | 114.8 ± 12.6 | 76.1 ± 8.4 | 18.5 ± 2.0 | 21.2 ± 2.3 | 11.4 ± 1.3 | 19.8 ± 2.2 | 79.0 ± 8.7 | 91.0 ± 32.3 | 19.0 ± 4.7 | 1075.0 ± 239.8 | 0.1 ± 0.0 | 809.6 ± 101.4 | 338.8 ± 54.2 | 433.3 ± 59.4 | 116.5 ± 39.9 | 27720.0 ± 4790.0 | 12500.0 ± 2317.0 | 2772.7 ± 221.9 |
| 24 | Laoxin town | E112°79'89" N30°20'67" | 212.7 ± 76.6 | 210.4 ± 23.1 | 107.8 ± 11.9 | 16.8 ± 4.6 | 22.8 ± 8.2 | 73.3 ± 8.1 | 9.3 ± 1.0 | 52.6 ± 14.4 | 58.3 ± 19.5 | 18.0 ± 4.2 | 1910.0 ± 529.3 | 0.6 ± 0.2 | 605.6 ± 94.6 | 596.6 ± 112.3 | 1281.2 ± 239.3 | 88.8 ± 12.5 | 66920.0 ± 5710.0 | 40590.0 ± 6730.0 | 5716.5 ± 673.7 |
| 25 | Xiongkou town | E112°80'01" N30°24'18" | 173.6 ± 19.1 | 54.5 ± 20.2 | 41.4 ± 4.6 | 16.2 ± 1.8 | 11.4 ± 1.2 | 12.7 ± 1.4 | 12.2 ± 1.3 | 74.0 ± 8.1 | 72.5 ± 21.9 | 17.0 ± 5.5 | 1575.0 ± 295.7 | 1.0 ± 0.5 | 1488.2 ± 298.4 | 617.1 ± 100.4 | 3390.4 ± 798.2 | 97.7 ± 21.4 | 39570.0 ± 4730.0 | 6905.0 ± 487.9 | 1957.8 ± 381.4 |
| 26 | Xiongkou town | E112°81'40" N30°23'86" | 66.4 ± 7.3 | 114.2 ± 12.6 | 54.6 ± 16.9 | 19.7 ± 7.1 | 24.0 ± 2.6 | 10.9 ± 1.2 | 17.7 ± 5.5 | 70.9 ± 25.5 | 20.0 ± 4.9 | 4.0 ± 1.7 | 940.0 ± 218.4 | 1.0 ± 0.3 | 384.4 ± 127.1 | 757.9 ± 214.6 | 413.4 ± 39.4 | 91.1 ± 23.2 | 39550.0 ± 3090.0 | 22940.0 ± 3910.0 | 113.4 ± 33.2 |
| 27 | Zhouji Street | E112°79'24" N30°37'71" | 244.6 ± 75.8 | 273.3 ± 30.1 | 74.6 ± 8.2 | 33.1 ± 3.6 | 21.5 ± 6.7 | 12.4 ± 1.4 | 24.4 ± 2.7 | 49.9 ± 13.6 | 98.4 ± 21.8 | 14.0 ± 5.6 | 1810.0 ± 743.4 | 1.8 ± 0.7 | 668.8 ± 142.6 | 459.0 ± 111.3 | 390.9 ± 78.4 | 116.2 ± 24.6 | 51120.0 ± 4122.0 | 157390.0 ± 39820.0 | 5319.6 ± 218.7 |
| 28 | Zhouji Street | E112°78'10" N30°36'86" | 195.6 ± 21.5 | 301.0 ± 82.2 | 144.8 ± 15.9 | 35.0 ± 3.9 | 15.3 ± 1.7 | 12.2 ± 3.3 | 17.8 ± 2.0 | 50.9 ± 5.6 | 40.4 ± 14.9 | 11.0 ± 4.6 | 930.0 ± 98.9 | 0.3 ± 0.1 | 636.2 ± 87.2 | 370.3 ± 54.7 | 371.6 ± 48.2 | 63.2 ± 11.3 | 50560.0 ± 4290.0 | 19055.0 ± 6121.4 | 5259.1 ± 416.3 |
| 29 | Houhu Farm | E112°74'68" N30°39'79" | 89.8 ± 9.9 | 185.3 ± 20.4 | 43.7 ± 4.8 | 20.8 ± 2.3 | 23.1 ± 2.5 | 23.3 ± 2.6 | 53.9 ± 5.9 | 133.8 ± 14.7 | 29.3 ± 7.7 | 5.0 ± 1.3 | 335.0 ± 111.3 | 0.1 ± 0.0 | 625.4 ± 121.9 | 403.1 ± 67.3 | 239.8 ± 47.1 | 142.4 ± 21.5 | 33450.0 ± 6370.0 | 23875.0 ± 5783.1 | 1216.6 ± 213.2 |
| 30 | Houhu Farm | E112°76'77" N30°40'41" | 89.3 ± 9.8 | 116.5 ± 12.8 | 51.4 ± 3.1 | 43.0 ± 11.7 | 29.3 ± 3.2 | 20.1 ± 2.2 | 49.6 ± 3.0 | 141.4 ± 38.6 | 84.7 ± 23.5 | 7.0 ± 2.6 | 1655.0 ± 275.6 | 0.3 ± 0.1 | 679.2 ± 47.4 | 341.4 ± 33.8 | 261.0 ± 39.5 | 111.3 ± 9.8 | 36920.0 ± 2917.0 | 57385.0 ± 4893.2 | 1271.0 ± 189.7 |
| 31 | Haokou town | E112°56'53" N30°31'45" | 45.7 ± 5.0 | 93.1 ± 25.4 | 65.1 ± 7.2 | 32.9 ± 11.9 | 341.6 ± 37.6 | 11.2 ± 3.1 | 36.6 ± 4.0 | 38.6 ± 13.9 | 70.7 ± 11.2 | 4.0 ± 2.1 | 1450.0 ± 856.4 | 0.9 ± 0.4 | 1270.6 ± 342.1 | 524.6 ± 75.2 | 1002.9 ± 123.8 | 152.0 ± 34.1 | 59320.0 ± 4890.0 | 10703.8 ± 1877.5 | 4552.5 ± 397.1 |
| 32 | Haokou town | E112°68'31" N30°36'18" | 406.2 ± 44.7 | 196.9 ± 21.7 | 36.3 ± 4.0 | 18.8 ± 7.0 | 44.2 ± 4.9 | 14.7 ± 1.6 | 14.5 ± 1.6 | 133.9 ± 36.5 | 20.7 ± 7.3 | 7.0 ± 3.7 | 955.0 ± 241.2 | 0.1 ± 0.0 | 581.0 ± 48.8 | 414.6 ± 52.5 | 603.0 ± 86.3 | 87.8 ± 9.4 | 74130.0 ± 6498.0 | 64190.0 ± 7830.0 | 3291.9 ± 405.4 |
| 33 | Zhangjin town | E112°66'87" N30°17'50" | 63.7 ± 3.8 | 64.2 ± 7.1 | 36.5 ± 4.0 | 12.5 ± 1.4 | 19.7 ± 1.2 | 16.8 ± 1.8 | 48.2 ± 5.3 | 102.8 ± 11.3 | 36.7 ± 14.8 | 4.0 ± 1.5 | 1775.0 ± 544.0 | 0.1 ± 0.0 | 807.4 ± 90.6 | 408.2 ± 85.4 | 546.4 ± 97.3 | 180.9 ± 32.2 | 34010.0 ± 2890.0 | 14810.0 ± 1390.0 | 260.6 ± 41.3 |
| 34 | Zhangjin town | E112°68'55" N30°16'23" | 129.7 ± 46.7 | 139.2 ± 30.6 | 137.8 ± 15.2 | 28.0 ± 3.1 | 18.9 ± 6.8 | 32.5 ± 3.6 | 22.1 ± 2.4 | 65.1 ± 7.2 | 30.7 ± 10.3 | 5.0 ± 2.4 | 1120.0 ± 233.4 | 0.1 ± 0.0 | 1409.0 ± 207.5 | 1000.9 ± 102.9 | 639.0 ± 59.6 | 151.6 ± 21.4 | 41480. 0 ± 3920.0 | 10565.0 ± 996.8 | 3510.9 ± 414.2 |
| 35 | Longwan town | E112°67'93" N30°22'17" | 170.0 ± 46.4 | 526.7 ± 57.9 | 38.2 ± 4.2 | 17.1 ± 5.3 | 29.2 ± 8.0 | 22.2 ± 2.4 | 47.4 ± 5.2 | 112.9 ± 35.0 | 32.3 ± 12.1 | 20.5 ± 7.5 | 1730.0 ± 473.3 | 0.6 ± 0.3 | 409.8 ± 70.1 | 851.8 ± 202.7 | 304.1 ± 59.7 | 118.0 ± 11.9 | 71330.0 ± 5893.0 | 26275.0 ± 5783.4 | 5145.1 ± 389.6 |
| 36 | Longwan town | E112°71'65" N30°28'38" | 219.5 ± 24.1 | 146.8 ± 52.8 | 109.3 ± 12.0 | 18.8 ± 6.9 | 37.0 ± 4.1 | 14.3 ± 5.1 | 34.3 ± 3.8 | 192.5 ± 21.2 | 31.4 ± 9.6 | 12.0 ± 4.2 | 850.0 ± 231.1 | 1.9 ± 0.9 | 969.5 ± 139.6 | 482.1 ± 89.4 | 609.4 ± 156.2 | 104.5 ± 9.5 | 55660.0 ± 6390.0 | 33340.0 ± 4809.0 | 7265.6 ± 639.9 |
| 37 | Longwan town | E112°73'71" N30°28'78" | 319.1 ± 35.1 | 192.1 ± 21.1 | 113.0 ± 12.4 | 8.8 ± 0.5 | 26.9 ± 3.0 | 15.7 ± 1.7 | 41.5 ± 4.6 | 89.7 ± 5.4 | 62.6 ± 21.4 | 9.5 ± 3.7 | 1337.5 ± 561.7 | 0.3 ± 0.2 | 788.3 ± 69.0 | 650.6 ± 198.8 | 257.1 ± 36.8 | 86.6 ± 6.3 | 44770.0 ± 5390.1 | 5904.0 ± 782.5 | 879.9 ± 59.7 |
| 38 | Longwan town | E112°74'50" N30°27'53" | 235.7 ± 25.9 | 185.1 ± 66.6 | 40.7 ± 2.4 | 26.5 ± 2.9 | 13.3 ± 4.1 | 15.4 ± 5.5 | 19.7 ± 1.2 | 31.5 ± 3.5 | 44.3 ± 14.3 | 10.5 ± 2.9 | 1485.0 ± 412.9 | 8.3 ± 2.3 | 583.7 ± 178.5 | 447.4 ± 156.1 | 746.4 ± 189.3 | 200.3 ± 41.7 | 98340.0 ± 6728.0 | 212795.9 ± 3419.6 | 1181.3 ± 211.4 |

Hc,Head capsule;Gi,Gill;He,Hepatopancreas;Am,Abdominal muscle.

Table S2. The parameters of elemental determinations by GFAAS and AAS methods.

| element | Instrument | | | | | | |
|---|---|---|---|---|---|---|---|
| | | **graphite furnace heating procedure** | | | | | |
| | | dry | incinerate | atomization | clean | | |
| Pb | AA240Z | 120℃, 22s | 600℃, 15s | 1000℃, 5s | 2500℃, 2s | | |
| Cd | | 110℃, 20s | 300℃, 20s | 900℃, 3s | 2500℃, 3s | | |
| | | **operating conditions of instruments** | | | | | |
| | | wavelength | lamp current | transmission band | graphite tube type | background correction | |
| Pb | | 217.0nm | 90% | 0.5nm | Coated graphite tube | Zeeman | |
| Cd | | 228.8nm | 50% | 0.5nm | Coated graphite tube | Zeeman | |
| | AA240FS | wavelength | lamp current | slit | gas flow | combustion head height | |
| Cu | | 324.7nm | 75% | 0.5nm | 1.1 L/min | 7.0 mm | |
| | | Negative high voltage of Photomultiplier tube | lamp current | gas flow | absorption tube | deoxidizer | carrier liquid |
| As | AFS-3100 | 270V | 60mA | 400mL/min | heat (850℃) | 2% NaBH4 + 0.5% NaOH | 5% HCl |
| Hg | | 280V | 25mA | 400mL/min | heat (850℃) | 2% NaBH4 + 0.5% NaOH | 5% HCl |

Table S3. The values of the elements contents (μg $kg^{-1}$) determind by given methods in CRM , recovery values (%), RSD (%) and LOQ values (μg $kg^{-1}$).

| | Pb | Cd | As | Cu |
|---|---|---|---|---|
| CRM values (μg $kg^{-1}$) | 101.7 ± 3.8 | 318.2 ± 45.9 | 236.5 ± 21.4 | 2319.2 ± 195.3 |
| Recovery (Mean %) | 97.23 | 106.43 | 102.41 | 100.31 |
| RSD (%) | 3.76 | 2.37 | 3.19 | 1.16 |
| LOQ (μg $kg^{-1}$) | 0.04 | 0.9 | < 0.01 | 0.2 |

Table S4. EDI (mg $kg^{-1}$ $day^{-1}$) for children (20kg).

| Sample numbers | Pb | | Hg | | Cd | | As | | Cu | |
|---|---|---|---|---|---|---|---|---|---|---|
| | He | Am | He | Am | He | Am | He | Am | He | Am |
| 1 | $1.09\times10^{-6}$ | $1.00\times10^{-6}$ | $4.03\times10^{-7}$ | $2.74\times10^{-6}$ | $3.45\times10^{-5}$ | $4.56\times10^{-8}$ | $9.02\times10^{-7}$ | $2.63\times10^{-7}$ | $3.25\times10^{-4}$ | $3.45\times10^{-4}$ |
| 2 | $3.37\times10^{-6}$ | $8.25\times10^{-7}$ | $4.36\times10^{-7}$ | $1.86\times10^{-6}$ | $1.32\times10^{-5}$ | $2.24\times10^{-8}$ | $1.93\times10^{-6}$ | $3.32\times10^{-7}$ | $1.88\times10^{-4}$ | $2.21\times10^{-4}$ |
| 3 | $1.46\times10^{-6}$ | $2.42\times10^{-6}$ | $9.76\times10^{-7}$ | $8.77\times10^{-6}$ | $1.62\times10^{-5}$ | $7.11\times10^{-9}$ | $1.64\times10^{-7}$ | $3.44\times10^{-7}$ | $8.56\times10^{-4}$ | $3.50\times10^{-4}$ |
| 4 | $2.55\times10^{-6}$ | $7.26\times10^{-7}$ | $9.16\times10^{-7}$ | $7.24\times10^{-6}$ | $9.63\times10^{-5}$ | $2.14\times10^{-8}$ | $4.82\times10^{-7}$ | $1.85\times10^{-7}$ | $6.57\times10^{-4}$ | $1.13\times10^{-4}$ |
| 5 | $7.19\times10^{-7}$ | $1.65\times10^{-6}$ | $3.73\times10^{-7}$ | $3.86\times10^{-6}$ | $1.92\times10^{-5}$ | $2.26\times10^{-8}$ | $2.62\times10^{-7}$ | $3.03\times10^{-7}$ | $2.42\times10^{-4}$ | $3.06\times10^{-4}$ |
| 6 | $1.58\times10^{-6}$ | $1.25\times10^{-6}$ | $4.26\times10^{-7}$ | $2.96\times10^{-6}$ | $1.78\times10^{-5}$ | $2.19\times10^{-8}$ | $2.56\times10^{-7}$ | $2.59\times10^{-7}$ | $1.89\times10^{-4}$ | $6.48\times10^{-5}$ |
| 7 | $1.94\times10^{-6}$ | $1.51\times10^{-6}$ | $8.23\times10^{-7}$ | $1.04\times10^{-5}$ | $3.70\times10^{-5}$ | $6.30\times10^{-8}$ | $2.68\times10^{-7}$ | $2.12\times10^{-7}$ | $2.21\times10^{-3}$ | $2.34\times10^{-5}$ |
| 8 | $2.52\times10^{-6}$ | $1.80\times10^{-6}$ | $1.05\times10^{-6}$ | $8.84\times10^{-6}$ | $2.16\times10^{-5}$ | $7.11\times10^{-9}$ | $3.23\times10^{-7}$ | $2.03\times10^{-7}$ | $3.39\times10^{-4}$ | $4.72\times10^{-5}$ |
| 9 | $2.33\times10^{-6}$ | $2.47\times10^{-6}$ | $2.05\times10^{-6}$ | $6.61\times10^{-6}$ | $3.08\times10^{-5}$ | $6.79\times10^{-8}$ | $5.00\times10^{-7}$ | $4.41\times10^{-7}$ | $1.16\times10^{-3}$ | $3.96\times10^{-4}$ |
| 10 | $7.16\times10^{-7}$ | $1.45\times10^{-6}$ | $1.00\times10^{-6}$ | $2.23\times10^{-6}$ | $7.48\times10^{-5}$ | $1.12\times10^{-7}$ | $1.98\times10^{-7}$ | $2.68\times10^{-7}$ | $3.27\times10^{-4}$ | $1.01\times10^{-4}$ |
| 11 | $1.47\times10^{-6}$ | $1.53\times10^{-6}$ | $1.24\times10^{-6}$ | $6.32\times10^{-6}$ | $5.50\times10^{-5}$ | $2.18\times10^{-8}$ | $2.79\times10^{-7}$ | $1.89\times10^{-7}$ | $2.14\times10^{-3}$ | $2.73\times10^{-5}$ |
| 12 | $1.45\times10^{-6}$ | $9.04\times10^{-7}$ | $1.10\times10^{-6}$ | $6.15\times10^{-6}$ | $2.40\times10^{-5}$ | $2.19\times10^{-8}$ | $4.38\times10^{-7}$ | $1.95\times10^{-7}$ | $7.66\times10^{-4}$ | $2.95\times10^{-4}$ |
| 13 | $1.72\times10^{-6}$ | $1.66\times10^{-6}$ | $1.77\times10^{-6}$ | $9.97\times10^{-6}$ | $5.55\times10^{-5}$ | $2.73\times10^{-7}$ | $2.40\times10^{-7}$ | $2.13\times10^{-7}$ | $5.24\times10^{-4}$ | $3.60\times10^{-5}$ |
| 14 | $1.14\times10^{-6}$ | $1.45\times10^{-6}$ | $1.07\times10^{-6}$ | $6.26\times10^{-6}$ | $5.86\times10^{-5}$ | $2.27\times10^{-8}$ | $3.47\times10^{-7}$ | $3.54\times10^{-7}$ | $6.19\times10^{-4}$ | $3.92\times10^{-5}$ |
| 15 | $1.79\times10^{-6}$ | $3.00\times10^{-6}$ | $9.09\times10^{-7}$ | $3.15\times10^{-6}$ | $4.03\times10^{-5}$ | $4.51\times10^{-8}$ | $2.11\times10^{-7}$ | $2.75\times10^{-7}$ | $2.50\times10^{-3}$ | $3.42\times10^{-4}$ |
| 16 | $1.88\times10^{-6}$ | $1.23\times10^{-6}$ | $1.01\times10^{-6}$ | $1.62\times10^{-5}$ | $3.60\times10^{-5}$ | $2.28\times10^{-8}$ | $2.63\times10^{-7}$ | $2.67\times10^{-7}$ | $3.79\times10^{-4}$ | $2.68\times10^{-4}$ |
| 17 | $2.22\times10^{-6}$ | $2.21\times10^{-6}$ | $5.50\times10^{-7}$ | $3.32\times10^{-6}$ | $2.20\times10^{-5}$ | $4.33\times10^{-8}$ | $1.12\times10^{-6}$ | $2.00\times10^{-7}$ | $4.97\times10^{-4}$ | $3.68\times10^{-5}$ |
| 18 | $1.97\times10^{-6}$ | $2.14\times10^{-6}$ | $1.02\times10^{-6}$ | $4.19\times10^{-6}$ | $1.85\times10^{-5}$ | $2.08\times10^{-8}$ | $8.36\times10^{-7}$ | $2.72\times10^{-7}$ | $3.54\times10^{-4}$ | $4.18\times10^{-5}$ |
| 19 | $5.06\times10^{-6}$ | $4.20\times10^{-6}$ | $9.46\times10^{-7}$ | $5.65\times10^{-6}$ | $3.31\times10^{-5}$ | $4.31\times10^{-8}$ | $6.45\times10^{-7}$ | $3.09\times10^{-7}$ | $3.02\times10^{-3}$ | $3.33\times10^{-4}$ |
| 20 | $6.75\times10^{-6}$ | $2.16\times10^{-6}$ | $7.73\times10^{-7}$ | $4.40\times10^{-6}$ | $2.31\times10^{-5}$ | $4.37\times10^{-8}$ | $1.73\times10^{-6}$ | $2.37\times10^{-7}$ | $7.84\times10^{-4}$ | $2.21\times10^{-5}$ |
| 21 | $2.31\times10^{-6}$ | $2.19\times10^{-6}$ | $9.89\times10^{-7}$ | $6.47\times10^{-6}$ | $6.01\times10^{-5}$ | $7.11\times10^{-9}$ | $1.55\times10^{-7}$ | $1.08\times10^{-7}$ | $5.89\times10^{-4}$ | $1.99\times10^{-4}$ |
| 22 | $2.48\times10^{-6}$ | $1.66\times10^{-6}$ | $1.23\times10^{-6}$ | $6.36\times10^{-6}$ | $6.48\times10^{-5}$ | $6.82\times10^{-8}$ | $5.16\times10^{-7}$ | $3.20\times10^{-7}$ | $2.13\times10^{-3}$ | $3.77\times10^{-4}$ |
| 23 | $2.53\times10^{-6}$ | $1.32\times10^{-6}$ | $6.59\times10^{-7}$ | $5.62\times10^{-6}$ | $3.58\times10^{-5}$ | $7.11\times10^{-9}$ | $4.33\times10^{-7}$ | $2.49\times10^{-7}$ | $4.16\times10^{-4}$ | $1.97\times10^{-4}$ |
| 24 | $3.59\times10^{-6}$ | $1.20\times10^{-6}$ | $3.10\times10^{-7}$ | $3.74\times10^{-6}$ | $6.36\times10^{-5}$ | $4.51\times10^{-8}$ | $1.28\times10^{-6}$ | $1.90\times10^{-7}$ | $1.35\times10^{-3}$ | $4.07\times10^{-4}$ |
| 25 | $1.38\times10^{-6}$ | $1.15\times10^{-6}$ | $4.06\times10^{-7}$ | $5.26\times10^{-6}$ | $5.25\times10^{-5}$ | $6.93\times10^{-8}$ | $3.39\times10^{-6}$ | $2.08\times10^{-7}$ | $2.30\times10^{-4}$ | $1.39\times10^{-4}$ |
| 26 | $1.82\times10^{-6}$ | $1.40\times10^{-6}$ | $5.89\times10^{-7}$ | $5.04\times10^{-6}$ | $3.13\times10^{-5}$ | $6.78\times10^{-8}$ | $4.13\times10^{-7}$ | $1.94\times10^{-7}$ | $7.64\times10^{-4}$ | $8.07\times10^{-6}$ |
| 27 | $2.48\times10^{-6}$ | $2.35\times10^{-6}$ | $8.13\times10^{-7}$ | $3.55\times10^{-6}$ | $6.03\times10^{-5}$ | $1.31\times10^{-7}$ | $3.91\times10^{-7}$ | $2.48\times10^{-7}$ | $5.24\times10^{-3}$ | $3.78\times10^{-4}$ |
| 28 | $4.82\times10^{-6}$ | $2.49\times10^{-6}$ | $5.93\times10^{-7}$ | $3.62\times10^{-6}$ | $3.10\times10^{-5}$ | $2.06\times10^{-8}$ | $3.71\times10^{-7}$ | $1.35\times10^{-7}$ | $6.35\times10^{-4}$ | $3.74\times10^{-4}$ |
| 29 | $1.46\times10^{-6}$ | $1.48\times10^{-6}$ | $1.80\times10^{-6}$ | $9.52\times10^{-6}$ | $1.12\times10^{-5}$ | $7.11\times10^{-9}$ | $2.40\times10^{-7}$ | $3.04\times10^{-7}$ | $7.95\times10^{-4}$ | $8.66\times10^{-5}$ |
| 30 | $1.71\times10^{-6}$ | $3.06\times10^{-6}$ | $1.65\times10^{-6}$ | $1.01\times10^{-5}$ | $5.51\times10^{-5}$ | $2.28\times10^{-8}$ | $2.61\times10^{-7}$ | $2.38\times10^{-7}$ | $1.91\times10^{-3}$ | $9.04\times10^{-5}$ |
| 31 | $2.17\times10^{-6}$ | $2.34\times10^{-6}$ | $1.22\times10^{-6}$ | $2.75\times10^{-6}$ | $4.83\times10^{-5}$ | $6.69\times10^{-8}$ | $1.00\times10^{-6}$ | $3.24\times10^{-7}$ | $3.56\times10^{-4}$ | $3.24\times10^{-4}$ |
| 32 | $1.21\times10^{-6}$ | $1.34\times10^{-6}$ | $4.83\times10^{-7}$ | $9.53\times10^{-6}$ | $3.18\times10^{-5}$ | $7.11\times10^{-9}$ | $6.02\times10^{-7}$ | $1.87\times10^{-7}$ | $2.14\times10^{-3}$ | $2.34\times10^{-4}$ |
| 33 | $1.22\times10^{-6}$ | $8.89\times10^{-7}$ | $1.61\times10^{-6}$ | $7.31\times10^{-6}$ | $5.91\times10^{-5}$ | $7.11\times10^{-9}$ | $5.46\times10^{-7}$ | $3.86\times10^{-7}$ | $4.93\times10^{-4}$ | $1.85\times10^{-5}$ |
| 34 | $4.59\times10^{-6}$ | $1.99\times10^{-6}$ | $7.36\times10^{-7}$ | $4.63\times10^{-6}$ | $3.73\times10^{-5}$ | $7.11\times10^{-9}$ | $6.38\times10^{-7}$ | $3.23\times10^{-7}$ | $3.52\times10^{-4}$ | $2.50\times10^{-4}$ |
| 35 | $1.27\times10^{-6}$ | $1.22\times10^{-6}$ | $1.58\times10^{-6}$ | $8.03\times10^{-6}$ | $5.76\times10^{-5}$ | $4.43\times10^{-8}$ | $3.04\times10^{-7}$ | $2.52\times10^{-7}$ | $8.75\times10^{-4}$ | $3.66\times10^{-4}$ |
| 36 | $3.64\times10^{-6}$ | $1.34\times10^{-6}$ | $1.14\times10^{-6}$ | $1.37\times10^{-5}$ | $2.83\times10^{-5}$ | $1.33\times10^{-7}$ | $6.09\times10^{-7}$ | $2.23\times10^{-7}$ | $1.11\times10^{-3}$ | $5.17\times10^{-4}$ |
| 37 | $3.76\times10^{-6}$ | $6.26\times10^{-7}$ | $1.38\times10^{-6}$ | $6.38\times10^{-6}$ | $4.45\times10^{-5}$ | $2.15\times10^{-8}$ | $2.57\times10^{-7}$ | $1.85\times10^{-7}$ | $1.97\times10^{-4}$ | $6.26\times10^{-5}$ |
| 38 | $1.36\times10^{-6}$ | $1.89\times10^{-6}$ | $6.56\times10^{-7}$ | $2.24\times10^{-6}$ | $4.95\times10^{-5}$ | $5.92\times10^{-7}$ | $7.46\times10^{-7}$ | $4.27\times10^{-7}$ | $7.09\times10^{-3}$ | $8.40\times10^{-5}$ |

Table S5. EDI (mg kg$^{-1}$ day$^{-1}$) for adults (70kg).

| Sample numbers | Pb | | Hg | | Cd | | As | | Cu | |
|---|---|---|---|---|---|---|---|---|---|---|
| | He | Am | He | Am | He | Am | He | Am | He | Am |
| 1 | $3.11\times10^{-7}$ | $2.87\times10^{-7}$ | $1.15\times10^{-7}$ | $7.83\times10^{-7}$ | $9.85\times10^{-6}$ | $1.30\times10^{-8}$ | $2.58\times10^{-7}$ | $7.51\times10^{-8}$ | $9.28\times10^{-5}$ | $9.86\times10^{-5}$ |
| 2 | $9.63\times10^{-7}$ | $2.36\times10^{-7}$ | $1.25\times10^{-7}$ | $5.31\times10^{-7}$ | $3.76\times10^{-6}$ | $6.39\times10^{-9}$ | $5.52\times10^{-7}$ | $9.50\times10^{-8}$ | $5.36\times10^{-5}$ | $6.31\times10^{-5}$ |
| 3 | $4.17\times10^{-7}$ | $6.91\times10^{-7}$ | $2.79\times10^{-7}$ | $2.51\times10^{-6}$ | $4.62\times10^{-6}$ | $2.03\times10^{-9}$ | $4.68\times10^{-8}$ | $9.84\times10^{-8}$ | $2.45\times10^{-4}$ | $9.99\times10^{-5}$ |
| 4 | $7.29\times10^{-7}$ | $2.07\times10^{-7}$ | $2.62\times10^{-7}$ | $2.07\times10^{-6}$ | $2.75\times10^{-5}$ | $6.12\times10^{-9}$ | $1.38\times10^{-7}$ | $5.29\times10^{-8}$ | $1.88\times10^{-4}$ | $3.24\times10^{-5}$ |
| 5 | $2.06\times10^{-7}$ | $4.72\times10^{-7}$ | $1.07\times10^{-7}$ | $1.10\times10^{-6}$ | $5.47\times10^{-6}$ | $6.46\times10^{-9}$ | $7.49\times10^{-8}$ | $8.67\times10^{-8}$ | $6.91\times10^{-5}$ | $8.74\times10^{-5}$ |
| 6 | $4.51\times10^{-7}$ | $3.58\times10^{-7}$ | $1.22\times10^{-7}$ | $8.46\times10^{-7}$ | $5.09\times10^{-6}$ | $6.27\times10^{-9}$ | $7.32\times10^{-8}$ | $7.40\times10^{-8}$ | $5.41\times10^{-5}$ | $1.85\times10^{-5}$ |
| 7 | $5.53\times10^{-7}$ | $4.31\times10^{-7}$ | $2.35\times10^{-7}$ | $2.96\times10^{-6}$ | $1.06\times10^{-5}$ | $1.80\times10^{-8}$ | $7.65\times10^{-8}$ | $6.06\times10^{-8}$ | $6.33\times10^{-4}$ | $6.68\times10^{-6}$ |
| 8 | $7.21\times10^{-7}$ | $5.14\times10^{-7}$ | $3.00\times10^{-7}$ | $2.52\times10^{-6}$ | $6.19\times10^{-6}$ | $2.03\times10^{-9}$ | $9.23\times10^{-8}$ | $5.79\times10^{-8}$ | $9.67\times10^{-5}$ | $1.35\times10^{-5}$ |
| 9 | $6.66\times10^{-7}$ | $7.05\times10^{-7}$ | $5.86\times10^{-7}$ | $1.89\times10^{-6}$ | $8.80\times10^{-6}$ | $1.94\times10^{-8}$ | $1.43\times10^{-7}$ | $1.26\times10^{-7}$ | $3.33\times10^{-4}$ | $1.13\times10^{-4}$ |
| 10 | $2.05\times10^{-7}$ | $4.15\times10^{-7}$ | $2.86\times10^{-7}$ | $6.36\times10^{-7}$ | $2.14\times10^{-5}$ | $3.19\times10^{-8}$ | $5.65\times10^{-8}$ | $7.66\times10^{-8}$ | $9.35\times10^{-5}$ | $2.88\times10^{-5}$ |
| 11 | $4.19\times10^{-7}$ | $4.37\times10^{-7}$ | $3.55\times10^{-7}$ | $1.81\times10^{-6}$ | $1.57\times10^{-5}$ | $6.23\times10^{-9}$ | $7.98\times10^{-8}$ | $5.41\times10^{-8}$ | $6.12\times10^{-4}$ | $7.79\times10^{-6}$ |
| 12 | $4.13\times10^{-7}$ | $2.58\times10^{-7}$ | $3.14\times10^{-7}$ | $1.76\times10^{-6}$ | $6.85\times10^{-6}$ | $6.27\times10^{-9}$ | $1.25\times10^{-7}$ | $5.56\times10^{-8}$ | $2.19\times10^{-4}$ | $8.43\times10^{-5}$ |
| 13 | $4.92\times10^{-7}$ | $4.74\times10^{-7}$ | $5.06\times10^{-7}$ | $2.85\times10^{-6}$ | $1.58\times10^{-5}$ | $7.79\times10^{-8}$ | $6.85\times10^{-8}$ | $6.09\times10^{-8}$ | $1.50\times10^{-4}$ | $1.03\times10^{-5}$ |
| 14 | $3.26\times10^{-7}$ | $4.15\times10^{-7}$ | $3.05\times10^{-7}$ | $1.79\times10^{-6}$ | $1.67\times10^{-5}$ | $6.48\times10^{-9}$ | $9.91\times10^{-8}$ | $1.01\times10^{-7}$ | $1.77\times10^{-4}$ | $1.12\times10^{-5}$ |
| 15 | $5.12\times10^{-7}$ | $8.56\times10^{-7}$ | $2.60\times10^{-7}$ | $9.00\times10^{-7}$ | $1.15\times10^{-5}$ | $1.29\times10^{-8}$ | $6.04\times10^{-8}$ | $7.85\times10^{-8}$ | $7.13\times10^{-4}$ | $9.77\times10^{-5}$ |
| 16 | $5.37\times10^{-7}$ | $3.52\times10^{-7}$ | $2.87\times10^{-7}$ | $4.64\times10^{-6}$ | $1.03\times10^{-5}$ | $6.51\times10^{-9}$ | $7.51\times10^{-8}$ | $7.63\times10^{-8}$ | $1.08\times10^{-4}$ | $7.67\times10^{-5}$ |
| 17 | $6.35\times10^{-7}$ | $6.30\times10^{-7}$ | $1.57\times10^{-7}$ | $9.47\times10^{-7}$ | $6.28\times10^{-6}$ | $1.24\times10^{-8}$ | $3.20\times10^{-7}$ | $5.73\times10^{-8}$ | $1.42\times10^{-4}$ | $1.05\times10^{-5}$ |
| 18 | $5.64\times10^{-7}$ | $6.12\times10^{-7}$ | $2.92\times10^{-7}$ | $1.20\times10^{-6}$ | $5.28\times10^{-6}$ | $5.93\times10^{-9}$ | $2.39\times10^{-7}$ | $7.78\times10^{-8}$ | $1.01\times10^{-4}$ | $1.19\times10^{-5}$ |
| 19 | $1.44\times10^{-6}$ | $1.20\times10^{-6}$ | $2.70\times10^{-7}$ | $1.61\times10^{-6}$ | $9.47\times10^{-6}$ | $1.23\times10^{-8}$ | $1.84\times10^{-7}$ | $8.84\times10^{-8}$ | $8.62\times10^{-4}$ | $9.51\times10^{-5}$ |
| 20 | $1.93\times10^{-6}$ | $6.18\times10^{-7}$ | $2.21\times10^{-7}$ | $1.26\times10^{-6}$ | $6.61\times10^{-6}$ | $1.25\times10^{-8}$ | $4.93\times10^{-7}$ | $6.77\times10^{-8}$ | $2.24\times10^{-4}$ | $6.31\times10^{-6}$ |
| 21 | $6.60\times10^{-7}$ | $6.26\times10^{-7}$ | $2.83\times10^{-7}$ | $1.85\times10^{-6}$ | $1.72\times10^{-5}$ | $2.03\times10^{-9}$ | $4.42\times10^{-8}$ | $3.10\times10^{-8}$ | $1.68\times10^{-4}$ | $5.68\times10^{-5}$ |
| 22 | $7.10\times10^{-7}$ | $4.74\times10^{-7}$ | $3.52\times10^{-7}$ | $1.82\times10^{-6}$ | $1.85\times10^{-5}$ | $1.95\times10^{-8}$ | $1.48\times10^{-7}$ | $9.14\times10^{-8}$ | $6.10\times10^{-4}$ | $1.08\times10^{-4}$ |
| 23 | $7.24\times10^{-7}$ | $3.76\times10^{-7}$ | $1.88\times10^{-7}$ | $1.61\times10^{-6}$ | $1.02\times10^{-5}$ | $2.03\times10^{-9}$ | $1.24\times10^{-7}$ | $7.11\times10^{-8}$ | $1.19\times10^{-4}$ | $5.64\times10^{-5}$ |
| 24 | $1.03\times10^{-6}$ | $3.41\times10^{-7}$ | $8.85\times10^{-8}$ | $1.07\times10^{-6}$ | $1.82\times10^{-5}$ | $1.29\times10^{-8}$ | $3.66\times10^{-7}$ | $5.42\times10^{-8}$ | $3.86\times10^{-4}$ | $1.16\times10^{-4}$ |
| 25 | $3.94\times10^{-7}$ | $3.29\times10^{-7}$ | $1.16\times10^{-7}$ | $1.50\times10^{-6}$ | $1.50\times10^{-5}$ | $1.98\times10^{-8}$ | $9.68\times10^{-7}$ | $5.96\times10^{-8}$ | $6.57\times10^{-5}$ | $3.98\times10^{-5}$ |
| 26 | $5.20\times10^{-7}$ | $4.00\times10^{-7}$ | $1.68\times10^{-7}$ | $1.44\times10^{-6}$ | $8.94\times10^{-6}$ | $1.94\times10^{-8}$ | $1.18\times10^{-7}$ | $5.56\times10^{-8}$ | $2.18\times10^{-4}$ | $2.31\times10^{-6}$ |
| 27 | $7.10\times10^{-7}$ | $6.73\times10^{-7}$ | $2.32\times10^{-7}$ | $1.01\times10^{-6}$ | $1.72\times10^{-5}$ | $3.74\times10^{-8}$ | $1.12\times10^{-7}$ | $7.08\times10^{-8}$ | $1.50\times10^{-3}$ | $1.08\times10^{-4}$ |
| 28 | $1.38\times10^{-6}$ | $7.11\times10^{-7}$ | $1.69\times10^{-7}$ | $1.03\times10^{-6}$ | $8.85\times10^{-6}$ | $5.88\times10^{-9}$ | $1.06\times10^{-7}$ | $3.86\times10^{-8}$ | $1.81\times10^{-4}$ | $1.07\times10^{-4}$ |
| 29 | $4.16\times10^{-7}$ | $4.23\times10^{-7}$ | $5.13\times10^{-7}$ | $2.72\times10^{-6}$ | $3.19\times10^{-6}$ | $2.03\times10^{-9}$ | $6.85\times10^{-8}$ | $8.68\times10^{-8}$ | $2.27\times10^{-4}$ | $2.47\times10^{-5}$ |
| 30 | $4.89\times10^{-7}$ | $8.74\times10^{-7}$ | $4.72\times10^{-7}$ | $2.87\times10^{-6}$ | $1.57\times10^{-5}$ | $6.52\times10^{-9}$ | $7.45\times10^{-8}$ | $6.79\times10^{-8}$ | $5.46\times10^{-4}$ | $2.58\times10^{-5}$ |
| 31 | $6.19\times10^{-7}$ | $6.69\times10^{-7}$ | $3.48\times10^{-7}$ | $7.85\times10^{-7}$ | $1.38\times10^{-5}$ | $1.91\times10^{-8}$ | $2.86\times10^{-7}$ | $9.27\times10^{-8}$ | $1.02\times10^{-4}$ | $9.25\times10^{-5}$ |
| 32 | $3.45\times10^{-7}$ | $3.82\times10^{-7}$ | $1.38\times10^{-7}$ | $2.72\times10^{-6}$ | $9.09\times10^{-6}$ | $2.03\times10^{-9}$ | $1.72\times10^{-7}$ | $5.35\times10^{-8}$ | $6.11\times10^{-4}$ | $6.69\times10^{-5}$ |
| 33 | $3.47\times10^{-7}$ | $2.54\times10^{-7}$ | $4.59\times10^{-7}$ | $2.09\times10^{-6}$ | $1.69\times10^{-5}$ | $2.03\times10^{-9}$ | $1.56\times10^{-7}$ | $1.10\times10^{-7}$ | $1.41\times10^{-4}$ | $5.30\times10^{-6}$ |
| 34 | $1.31\times10^{-6}$ | $5.69\times10^{-7}$ | $2.10\times10^{-7}$ | $1.32\times10^{-6}$ | $1.07\times10^{-5}$ | $2.03\times10^{-9}$ | $1.82\times10^{-7}$ | $9.24\times10^{-8}$ | $1.01\times10^{-4}$ | $7.14\times10^{-5}$ |
| 35 | $3.64\times10^{-7}$ | $3.48\times10^{-7}$ | $4.51\times10^{-7}$ | $2.29\times10^{-6}$ | $1.65\times10^{-5}$ | $1.26\times10^{-8}$ | $8.68\times10^{-8}$ | $7.19\times10^{-8}$ | $2.50\times10^{-4}$ | $1.05\times10^{-4}$ |
| 36 | $1.04\times10^{-6}$ | $3.82\times10^{-7}$ | $3.26\times10^{-7}$ | $3.91\times10^{-6}$ | $8.09\times10^{-6}$ | $3.81\times10^{-8}$ | $1.74\times10^{-7}$ | $6.37\times10^{-8}$ | $3.17\times10^{-4}$ | $1.48\times10^{-4}$ |
| 37 | $1.08\times10^{-6}$ | $1.79\times10^{-7}$ | $3.95\times10^{-7}$ | $1.82\times10^{-6}$ | $1.27\times10^{-5}$ | $6.14\times10^{-9}$ | $7.34\times10^{-8}$ | $5.28\times10^{-8}$ | $5.62\times10^{-5}$ | $1.79\times10^{-5}$ |
| 38 | $3.87\times10^{-7}$ | $5.39\times10^{-7}$ | $1.87\times10^{-7}$ | $6.40\times10^{-7}$ | $1.41\times10^{-5}$ | $1.69\times10^{-7}$ | $2.13\times10^{-7}$ | $1.22\times10^{-7}$ | $2.02\times10^{-3}$ | $2.40\times10^{-5}$ |

Table S6. Target hazard quotient (THQ) of five toxic elements in children (20kg).

| Sample numbers | Pb | | Hg | | Cd | | As | | Cu | |
|---|---|---|---|---|---|---|---|---|---|---|
| | He | Am | He | Am | He | Am | He | Am | He | Am |
| 1 | $1.53\times10^{-6}$ | $1.41\times10^{-6}$ | $1.14\times10^{-7}$ | $7.72\times10^{-7}$ | $9.71\times10^{-4}$ | $1.29\times10^{-6}$ | $8.48\times10^{-5}$ | $2.47\times10^{-5}$ | $2.29\times10^{-4}$ | $2.43\times10^{-4}$ |
| 2 | $4.75\times10^{-6}$ | $1.16\times10^{-6}$ | $1.23\times10^{-7}$ | $5.23\times10^{-7}$ | $3.71\times10^{-4}$ | $6.31\times10^{-7}$ | $1.81\times10^{-4}$ | $3.12\times10^{-5}$ | $1.32\times10^{-4}$ | $1.56\times10^{-4}$ |
| 3 | $2.06\times10^{-6}$ | $3.41\times10^{-6}$ | $2.75\times10^{-7}$ | $2.47\times10^{-6}$ | $4.55\times10^{-4}$ | $2.00\times10^{-7}$ | $1.54\times10^{-5}$ | $3.24\times10^{-5}$ | $6.03\times10^{-4}$ | $2.46\times10^{-4}$ |
| 4 | $3.59\times10^{-6}$ | $1.02\times10^{-6}$ | $2.58\times10^{-7}$ | $2.04\times10^{-6}$ | $2.71\times10^{-3}$ | $6.03\times10^{-7}$ | $4.53\times10^{-5}$ | $1.74\times10^{-5}$ | $4.63\times10^{-4}$ | $7.98\times10^{-5}$ |
| 5 | $1.01\times10^{-6}$ | $2.33\times10^{-6}$ | $1.05\times10^{-7}$ | $1.09\times10^{-6}$ | $5.40\times10^{-4}$ | $6.37\times10^{-7}$ | $2.46\times10^{-5}$ | $2.85\times10^{-5}$ | $1.70\times10^{-4}$ | $2.15\times10^{-4}$ |
| 6 | $2.22\times10^{-6}$ | $1.76\times10^{-6}$ | $1.20\times10^{-7}$ | $8.34\times10^{-7}$ | $5.02\times10^{-4}$ | $6.18\times10^{-7}$ | $2.41\times10^{-5}$ | $2.43\times10^{-5}$ | $1.33\times10^{-4}$ | $4.57\times10^{-5}$ |
| 7 | $2.73\times10^{-6}$ | $2.13\times10^{-6}$ | $2.32\times10^{-7}$ | $2.92\times10^{-6}$ | $1.04\times10^{-3}$ | $1.77\times10^{-6}$ | $2.52\times10^{-5}$ | $1.99\times10^{-5}$ | $1.56\times10^{-3}$ | $1.65\times10^{-5}$ |
| 8 | $3.56\times10^{-6}$ | $2.54\times10^{-6}$ | $2.96\times10^{-7}$ | $2.49\times10^{-6}$ | $6.10\times10^{-4}$ | $2.00\times10^{-7}$ | $3.03\times10^{-5}$ | $1.90\times10^{-5}$ | $2.39\times10^{-4}$ | $3.32\times10^{-5}$ |
| 9 | $3.28\times10^{-6}$ | $3.48\times10^{-6}$ | $5.78\times10^{-7}$ | $1.86\times10^{-6}$ | $8.68\times10^{-4}$ | $1.91\times10^{-6}$ | $4.69\times10^{-5}$ | $4.14\times10^{-5}$ | $8.20\times10^{-4}$ | $2.79\times10^{-4}$ |
| 10 | $1.01\times10^{-6}$ | $2.04\times10^{-6}$ | $2.82\times10^{-7}$ | $6.28\times10^{-7}$ | $2.11\times10^{-3}$ | $3.15\times10^{-6}$ | $1.86\times10^{-5}$ | $2.52\times10^{-5}$ | $2.31\times10^{-4}$ | $7.11\times10^{-5}$ |
| 11 | $2.06\times10^{-6}$ | $2.16\times10^{-6}$ | $3.50\times10^{-7}$ | $1.78\times10^{-6}$ | $1.55\times10^{-3}$ | $6.14\times10^{-7}$ | $2.62\times10^{-5}$ | $1.78\times10^{-5}$ | $1.51\times10^{-3}$ | $1.92\times10^{-5}$ |
| 12 | $2.04\times10^{-6}$ | $1.27\times10^{-6}$ | $3.10\times10^{-7}$ | $1.73\times10^{-6}$ | $6.76\times10^{-4}$ | $6.18\times10^{-7}$ | $4.11\times10^{-5}$ | $1.83\times10^{-5}$ | $5.40\times10^{-4}$ | $2.08\times10^{-4}$ |
| 13 | $2.43\times10^{-6}$ | $2.34\times10^{-6}$ | $4.99\times10^{-7}$ | $2.81\times10^{-6}$ | $1.56\times10^{-3}$ | $7.69\times10^{-6}$ | $2.25\times10^{-5}$ | $2.00\times10^{-5}$ | $3.69\times10^{-4}$ | $2.54\times10^{-5}$ |
| 14 | $1.61\times10^{-6}$ | $2.04\times10^{-6}$ | $3.00\times10^{-7}$ | $1.76\times10^{-6}$ | $1.65\times10^{-3}$ | $6.39\times10^{-7}$ | $3.26\times10^{-5}$ | $3.33\times10^{-5}$ | $4.36\times10^{-4}$ | $2.76\times10^{-5}$ |
| 15 | $2.52\times10^{-6}$ | $4.22\times10^{-6}$ | $2.56\times10^{-7}$ | $8.88\times10^{-7}$ | $1.14\times10^{-3}$ | $1.27\times10^{-6}$ | $1.99\times10^{-5}$ | $2.58\times10^{-5}$ | $1.76\times10^{-3}$ | $2.41\times10^{-4}$ |
| 16 | $2.65\times10^{-6}$ | $1.73\times10^{-6}$ | $2.83\times10^{-7}$ | $4.58\times10^{-6}$ | $1.01\times10^{-3}$ | $6.42\times10^{-7}$ | $2.47\times10^{-5}$ | $2.51\times10^{-5}$ | $2.67\times10^{-4}$ | $1.89\times10^{-4}$ |
| 17 | $3.13\times10^{-6}$ | $3.11\times10^{-6}$ | $1.55\times10^{-7}$ | $9.34\times10^{-7}$ | $6.19\times10^{-4}$ | $1.22\times10^{-6}$ | $1.05\times10^{-4}$ | $1.88\times10^{-5}$ | $3.50\times10^{-4}$ | $2.60\times10^{-5}$ |
| 18 | $2.78\times10^{-6}$ | $3.02\times10^{-6}$ | $2.88\times10^{-7}$ | $1.18\times10^{-6}$ | $5.21\times10^{-4}$ | $5.85\times10^{-7}$ | $7.85\times10^{-5}$ | $2.56\times10^{-5}$ | $2.49\times10^{-4}$ | $2.95\times10^{-5}$ |
| 19 | $7.12\times10^{-6}$ | $5.92\times10^{-6}$ | $2.67\times10^{-7}$ | $1.59\times10^{-6}$ | $9.34\times10^{-4}$ | $1.22\times10^{-6}$ | $6.06\times10^{-5}$ | $2.91\times10^{-5}$ | $2.12\times10^{-3}$ | $2.34\times10^{-4}$ |
| 20 | $9.51\times10^{-6}$ | $3.05\times10^{-6}$ | $2.18\times10^{-7}$ | $1.24\times10^{-6}$ | $6.52\times10^{-4}$ | $1.23\times10^{-6}$ | $1.62\times10^{-4}$ | $2.23\times10^{-5}$ | $5.52\times10^{-4}$ | $1.56\times10^{-5}$ |
| 21 | $3.26\times10^{-6}$ | $3.09\times10^{-6}$ | $2.79\times10^{-7}$ | $1.82\times10^{-6}$ | $1.69\times10^{-3}$ | $2.00\times10^{-7}$ | $1.45\times10^{-5}$ | $1.02\times10^{-5}$ | $4.15\times10^{-4}$ | $1.40\times10^{-4}$ |
| 22 | $3.50\times10^{-6}$ | $2.34\times10^{-6}$ | $3.47\times10^{-7}$ | $1.79\times10^{-6}$ | $1.83\times10^{-3}$ | $1.92\times10^{-6}$ | $4.85\times10^{-5}$ | $3.01\times10^{-5}$ | $1.50\times10^{-3}$ | $2.65\times10^{-4}$ |
| 23 | $3.57\times10^{-6}$ | $1.85\times10^{-6}$ | $1.86\times10^{-7}$ | $1.58\times10^{-6}$ | $1.01\times10^{-3}$ | $2.00\times10^{-7}$ | $4.07\times10^{-5}$ | $2.34\times10^{-5}$ | $2.93\times10^{-4}$ | $1.39\times10^{-4}$ |
| 24 | $5.06\times10^{-6}$ | $1.68\times10^{-6}$ | $8.73\times10^{-8}$ | $1.05\times10^{-6}$ | $1.79\times10^{-3}$ | $1.27\times10^{-6}$ | $1.20\times10^{-4}$ | $1.78\times10^{-5}$ | $9.52\times10^{-4}$ | $2.87\times10^{-4}$ |
| 25 | $1.94\times10^{-6}$ | $1.62\times10^{-6}$ | $1.15\times10^{-7}$ | $1.48\times10^{-6}$ | $1.48\times10^{-3}$ | $1.95\times10^{-6}$ | $3.18\times10^{-4}$ | $1.96\times10^{-5}$ | $1.62\times10^{-4}$ | $9.81\times10^{-5}$ |
| 26 | $2.56\times10^{-6}$ | $1.97\times10^{-6}$ | $1.66\times10^{-7}$ | $1.42\times10^{-6}$ | $8.82\times10^{-4}$ | $1.91\times10^{-6}$ | $3.88\times10^{-5}$ | $1.83\times10^{-5}$ | $5.38\times10^{-4}$ | $5.69\times10^{-6}$ |
| 27 | $3.50\times10^{-6}$ | $3.32\times10^{-6}$ | $2.29\times10^{-7}$ | $1.00\times10^{-6}$ | $1.70\times10^{-3}$ | $3.68\times10^{-6}$ | $3.67\times10^{-5}$ | $2.33\times10^{-5}$ | $3.69\times10^{-3}$ | $2.67\times10^{-4}$ |
| 28 | $6.80\times10^{-6}$ | $3.51\times10^{-6}$ | $1.67\times10^{-7}$ | $1.02\times10^{-6}$ | $8.73\times10^{-4}$ | $5.80\times10^{-7}$ | $3.49\times10^{-5}$ | $1.27\times10^{-5}$ | $4.47\times10^{-4}$ | $2.64\times10^{-4}$ |
| 29 | $2.05\times10^{-6}$ | $2.09\times10^{-6}$ | $5.06\times10^{-7}$ | $2.68\times10^{-6}$ | $3.14\times10^{-4}$ | $2.00\times10^{-7}$ | $2.25\times10^{-5}$ | $2.86\times10^{-5}$ | $5.60\times10^{-4}$ | $6.10\times10^{-5}$ |
| 30 | $2.41\times10^{-6}$ | $4.31\times10^{-6}$ | $4.66\times10^{-7}$ | $2.83\times10^{-6}$ | $1.55\times10^{-3}$ | $6.43\times10^{-7}$ | $2.45\times10^{-5}$ | $2.23\times10^{-5}$ | $1.35\times10^{-3}$ | $6.37\times10^{-5}$ |
| 31 | $3.05\times10^{-6}$ | $3.30\times10^{-6}$ | $3.44\times10^{-7}$ | $7.74\times10^{-7}$ | $1.36\times10^{-3}$ | $1.89\times10^{-6}$ | $9.41\times10^{-5}$ | $3.05\times10^{-5}$ | $2.51\times10^{-4}$ | $2.28\times10^{-4}$ |
| 32 | $1.70\times10^{-6}$ | $1.88\times10^{-6}$ | $1.36\times10^{-7}$ | $2.68\times10^{-6}$ | $8.96\times10^{-4}$ | $2.00\times10^{-7}$ | $5.66\times10^{-5}$ | $1.76\times10^{-5}$ | $1.51\times10^{-3}$ | $1.65\times10^{-4}$ |
| 33 | $1.71\times10^{-6}$ | $1.25\times10^{-6}$ | $4.52\times10^{-7}$ | $2.06\times10^{-6}$ | $1.67\times10^{-3}$ | $2.00\times10^{-7}$ | $5.13\times10^{-5}$ | $3.63\times10^{-5}$ | $3.47\times10^{-4}$ | $1.31\times10^{-5}$ |
| 34 | $6.47\times10^{-6}$ | $2.81\times10^{-6}$ | $2.07\times10^{-7}$ | $1.31\times10^{-6}$ | $1.05\times10^{-3}$ | $2.00\times10^{-7}$ | $6.00\times10^{-5}$ | $3.04\times10^{-5}$ | $2.48\times10^{-4}$ | $1.76\times10^{-4}$ |
| 35 | $1.79\times10^{-6}$ | $1.71\times10^{-6}$ | $4.45\times10^{-7}$ | $2.26\times10^{-6}$ | $1.62\times10^{-3}$ | $1.25\times10^{-6}$ | $2.85\times10^{-5}$ | $2.37\times10^{-5}$ | $6.17\times10^{-4}$ | $2.58\times10^{-4}$ |
| 36 | $5.13\times10^{-6}$ | $1.88\times10^{-6}$ | $3.22\times10^{-7}$ | $3.86\times10^{-6}$ | $7.98\times10^{-4}$ | $3.76\times10^{-6}$ | $5.72\times10^{-5}$ | $2.09\times10^{-5}$ | $7.82\times10^{-4}$ | $3.64\times10^{-4}$ |
| 37 | $5.30\times10^{-6}$ | $8.82\times10^{-7}$ | $3.89\times10^{-7}$ | $1.80\times10^{-6}$ | $1.26\times10^{-3}$ | $6.05\times10^{-7}$ | $2.41\times10^{-5}$ | $1.74\times10^{-5}$ | $1.39\times10^{-4}$ | $4.41\times10^{-5}$ |
| 38 | $1.91\times10^{-6}$ | $2.66\times10^{-6}$ | $1.85\times10^{-7}$ | $6.32\times10^{-7}$ | $1.39\times10^{-3}$ | $1.67\times10^{-5}$ | $7.00\times10^{-5}$ | $4.02\times10^{-5}$ | $4.99\times10^{-3}$ | $5.92\times10^{-5}$ |

Table S7. Target hazard quotient (THQ) of five toxic elements in adults (70kg).

| Sample numbers | Pb | | Hg | | Cd | | As | | Cu | |
|---|---|---|---|---|---|---|---|---|---|---|
| | He | Am | He | Am | He | Am | He | Am | He | Am |
| 1 | $5.70\times10^{-6}$ | $5.25\times10^{-6}$ | $4.22\times10^{-7}$ | $2.87\times10^{-6}$ | $3.61\times10^{-3}$ | $4.77\times10^{-6}$ | $3.15\times10^{-4}$ | $9.18\times10^{-5}$ | $8.50\times10^{-4}$ | $9.03\times10^{-4}$ |
| 2 | $1.76\times10^{-5}$ | $4.32\times10^{-6}$ | $4.57\times10^{-7}$ | $1.94\times10^{-6}$ | $1.38\times10^{-3}$ | $2.34\times10^{-6}$ | $6.74\times10^{-4}$ | $1.16\times10^{-4}$ | $4.91\times10^{-4}$ | $5.78\times10^{-4}$ |
| 3 | $7.63\times10^{-6}$ | $1.27\times10^{-5}$ | $1.02\times10^{-6}$ | $9.18\times10^{-6}$ | $1.69\times10^{-3}$ | $7.45\times10^{-7}$ | $5.71\times10^{-5}$ | $1.20\times10^{-4}$ | $2.24\times10^{-3}$ | $9.15\times10^{-4}$ |
| 4 | $1.34\times10^{-5}$ | $3.80\times10^{-6}$ | $9.59\times10^{-7}$ | $7.57\times10^{-6}$ | $1.01\times10^{-2}$ | $2.24\times10^{-6}$ | $1.68\times10^{-4}$ | $6.46\times10^{-5}$ | $1.72\times10^{-3}$ | $2.97\times10^{-4}$ |
| 5 | $3.76\times10^{-6}$ | $8.64\times10^{-6}$ | $3.90\times10^{-7}$ | $4.04\times10^{-6}$ | $2.00\times10^{-3}$ | $2.37\times10^{-6}$ | $9.14\times10^{-5}$ | $1.06\times10^{-4}$ | $6.33\times10^{-4}$ | $8.00\times10^{-4}$ |
| 6 | $8.26\times10^{-6}$ | $6.55\times10^{-6}$ | $4.46\times10^{-7}$ | $3.10\times10^{-6}$ | $1.87\times10^{-3}$ | $2.30\times10^{-6}$ | $8.94\times10^{-5}$ | $9.04\times10^{-5}$ | $4.95\times10^{-4}$ | $1.70\times10^{-4}$ |
| 7 | $1.01\times10^{-5}$ | $7.89\times10^{-6}$ | $8.61\times10^{-7}$ | $1.08\times10^{-5}$ | $3.87\times10^{-3}$ | $6.59\times10^{-6}$ | $9.34\times10^{-5}$ | $7.40\times10^{-5}$ | $5.80\times10^{-3}$ | $6.12\times10^{-5}$ |
| 8 | $1.32\times10^{-5}$ | $9.42\times10^{-6}$ | $1.10\times10^{-6}$ | $9.25\times10^{-6}$ | $2.27\times10^{-3}$ | $7.45\times10^{-7}$ | $1.13\times10^{-4}$ | $7.07\times10^{-5}$ | $8.86\times10^{-4}$ | $1.23\times10^{-4}$ |
| 9 | $1.22\times10^{-5}$ | $1.29\times10^{-5}$ | $2.15\times10^{-6}$ | $6.92\times10^{-6}$ | $3.22\times10^{-3}$ | $7.11\times10^{-6}$ | $1.74\times10^{-4}$ | $1.54\times10^{-4}$ | $3.05\times10^{-3}$ | $1.04\times10^{-3}$ |
| 10 | $3.75\times10^{-6}$ | $7.60\times10^{-6}$ | $1.05\times10^{-6}$ | $2.33\times10^{-6}$ | $7.83\times10^{-3}$ | $1.17\times10^{-5}$ | $6.90\times10^{-5}$ | $9.35\times10^{-5}$ | $8.56\times10^{-4}$ | $2.64\times10^{-4}$ |
| 11 | $7.67\times10^{-6}$ | $8.01\times10^{-6}$ | $1.30\times10^{-6}$ | $6.62\times10^{-6}$ | $5.75\times10^{-3}$ | $2.28\times10^{-6}$ | $9.75\times10^{-5}$ | $6.60\times10^{-5}$ | $5.60\times10^{-3}$ | $7.14\times10^{-5}$ |
| 12 | $7.56\times10^{-6}$ | $4.73\times10^{-6}$ | $1.15\times10^{-6}$ | $6.43\times10^{-6}$ | $2.51\times10^{-3}$ | $2.30\times10^{-6}$ | $1.53\times10^{-4}$ | $6.79\times10^{-5}$ | $2.00\times10^{-3}$ | $7.72\times10^{-4}$ |
| 13 | $9.01\times10^{-6}$ | $8.68\times10^{-6}$ | $1.85\times10^{-6}$ | $1.04\times10^{-5}$ | $5.80\times10^{-3}$ | $2.85\times10^{-5}$ | $8.36\times10^{-5}$ | $7.43\times10^{-5}$ | $1.37\times10^{-3}$ | $9.42\times10^{-5}$ |
| 14 | $5.98\times10^{-6}$ | $7.60\times10^{-6}$ | $1.12\times10^{-6}$ | $6.55\times10^{-6}$ | $6.14\times10^{-3}$ | $2.37\times10^{-6}$ | $1.21\times10^{-4}$ | $1.24\times10^{-4}$ | $1.62\times10^{-3}$ | $1.02\times10^{-4}$ |
| 15 | $9.38\times10^{-6}$ | $1.57\times10^{-5}$ | $9.52\times10^{-7}$ | $3.30\times10^{-6}$ | $4.22\times10^{-3}$ | $4.72\times10^{-6}$ | $7.37\times10^{-5}$ | $9.58\times10^{-5}$ | $6.53\times10^{-3}$ | $8.95\times10^{-4}$ |
| 16 | $9.83\times10^{-6}$ | $6.44\times10^{-6}$ | $1.05\times10^{-6}$ | $1.70\times10^{-5}$ | $3.76\times10^{-3}$ | $2.38\times10^{-6}$ | $9.17\times10^{-5}$ | $9.32\times10^{-5}$ | $9.92\times10^{-4}$ | $7.03\times10^{-4}$ |
| 17 | $1.16\times10^{-5}$ | $1.15\times10^{-5}$ | $5.75\times10^{-7}$ | $3.47\times10^{-6}$ | $2.30\times10^{-3}$ | $4.53\times10^{-6}$ | $3.91\times10^{-4}$ | $6.99\times10^{-5}$ | $1.30\times10^{-3}$ | $9.64\times10^{-5}$ |
| 18 | $1.03\times10^{-5}$ | $1.12\times10^{-5}$ | $1.07\times10^{-6}$ | $4.39\times10^{-6}$ | $1.93\times10^{-3}$ | $2.17\times10^{-6}$ | $2.92\times10^{-4}$ | $9.50\times10^{-5}$ | $9.25\times10^{-4}$ | $1.09\times10^{-4}$ |
| 19 | $2.65\times10^{-5}$ | $2.20\times10^{-5}$ | $9.90\times10^{-7}$ | $5.91\times10^{-6}$ | $3.47\times10^{-3}$ | $4.51\times10^{-6}$ | $2.25\times10^{-4}$ | $1.08\times10^{-4}$ | $7.89\times10^{-3}$ | $8.71\times10^{-4}$ |
| 20 | $3.53\times10^{-5}$ | $1.13\times10^{-5}$ | $8.09\times10^{-7}$ | $4.60\times10^{-6}$ | $2.42\times10^{-3}$ | $4.58\times10^{-6}$ | $6.02\times10^{-4}$ | $8.27\times10^{-5}$ | $2.05\times10^{-3}$ | $5.78\times10^{-5}$ |
| 21 | $1.21\times10^{-5}$ | $1.15\times10^{-5}$ | $1.04\times10^{-6}$ | $6.78\times10^{-6}$ | $6.29\times10^{-3}$ | $7.45\times10^{-7}$ | $5.40\times10^{-5}$ | $3.78\times10^{-5}$ | $1.54\times10^{-3}$ | $5.20\times10^{-4}$ |
| 22 | $1.30\times10^{-5}$ | $8.68\times10^{-6}$ | $1.29\times10^{-6}$ | $6.66\times10^{-6}$ | $6.78\times10^{-3}$ | $7.14\times10^{-6}$ | $1.80\times10^{-4}$ | $1.12\times10^{-4}$ | $5.58\times10^{-3}$ | $9.85\times10^{-4}$ |
| 23 | $1.33\times10^{-5}$ | $6.89\times10^{-6}$ | $6.90\times10^{-7}$ | $5.88\times10^{-6}$ | $3.75\times10^{-3}$ | $7.45\times10^{-7}$ | $1.51\times10^{-4}$ | $8.68\times10^{-5}$ | $1.09\times10^{-3}$ | $5.16\times10^{-4}$ |
| 24 | $1.88\times10^{-5}$ | $6.26\times10^{-6}$ | $3.24\times10^{-7}$ | $3.92\times10^{-6}$ | $6.66\times10^{-3}$ | $4.72\times10^{-6}$ | $4.47\times10^{-4}$ | $6.61\times10^{-5}$ | $3.54\times10^{-3}$ | $1.06\times10^{-3}$ |
| 25 | $7.22\times10^{-6}$ | $6.03\times10^{-6}$ | $4.25\times10^{-7}$ | $5.51\times10^{-6}$ | $5.49\times10^{-3}$ | $7.25\times10^{-6}$ | $1.18\times10^{-3}$ | $7.27\times10^{-5}$ | $6.02\times10^{-4}$ | $3.64\times10^{-4}$ |
| 26 | $9.52\times10^{-6}$ | $7.33\times10^{-6}$ | $6.17\times10^{-7}$ | $5.28\times10^{-6}$ | $3.28\times10^{-3}$ | $7.10\times10^{-6}$ | $1.44\times10^{-4}$ | $6.78\times10^{-5}$ | $2.00\times10^{-3}$ | $2.11\times10^{-5}$ |
| 27 | $1.30\times10^{-5}$ | $1.23\times10^{-5}$ | $8.51\times10^{-7}$ | $3.72\times10^{-6}$ | $6.31\times10^{-3}$ | $1.37\times10^{-5}$ | $1.36\times10^{-4}$ | $8.65\times10^{-5}$ | $1.37\times10^{-2}$ | $9.90\times10^{-4}$ |
| 28 | $2.52\times10^{-5}$ | $1.30\times10^{-5}$ | $6.21\times10^{-7}$ | $3.79\times10^{-6}$ | $3.24\times10^{-3}$ | $2.15\times10^{-6}$ | $1.30\times10^{-4}$ | $4.71\times10^{-5}$ | $1.66\times10^{-3}$ | $9.79\times10^{-4}$ |
| 29 | $7.62\times10^{-6}$ | $7.74\times10^{-6}$ | $1.88\times10^{-6}$ | $9.96\times10^{-6}$ | $1.17\times10^{-3}$ | $7.45\times10^{-7}$ | $8.36\times10^{-5}$ | $1.06\times10^{-4}$ | $2.08\times10^{-3}$ | $2.26\times10^{-4}$ |
| 30 | $8.96\times10^{-6}$ | $1.60\times10^{-5}$ | $1.73\times10^{-6}$ | $1.05\times10^{-5}$ | $5.77\times10^{-3}$ | $2.39\times10^{-6}$ | $9.10\times10^{-5}$ | $8.29\times10^{-5}$ | $5.00\times10^{-3}$ | $2.37\times10^{-4}$ |
| 31 | $1.13\times10^{-5}$ | $1.22\times10^{-5}$ | $1.28\times10^{-6}$ | $2.87\times10^{-6}$ | $5.05\times10^{-3}$ | $7.00\times10^{-6}$ | $3.50\times10^{-4}$ | $1.13\times10^{-4}$ | $9.33\times10^{-4}$ | $8.48\times10^{-4}$ |
| 32 | $6.33\times10^{-6}$ | $7.00\times10^{-6}$ | $5.05\times10^{-7}$ | $9.97\times10^{-6}$ | $3.33\times10^{-3}$ | $7.45\times10^{-7}$ | $2.10\times10^{-4}$ | $6.54\times10^{-5}$ | $5.59\times10^{-3}$ | $6.13\times10^{-4}$ |
| 33 | $6.36\times10^{-6}$ | $4.65\times10^{-6}$ | $1.68\times10^{-6}$ | $7.66\times10^{-6}$ | $6.19\times10^{-3}$ | $7.45\times10^{-7}$ | $1.90\times10^{-4}$ | $1.35\times10^{-4}$ | $1.29\times10^{-3}$ | $4.85\times10^{-5}$ |
| 34 | $2.40\times10^{-5}$ | $1.04\times10^{-5}$ | $7.70\times10^{-7}$ | $4.85\times10^{-6}$ | $3.90\times10^{-3}$ | $7.45\times10^{-7}$ | $2.23\times10^{-4}$ | $1.13\times10^{-4}$ | $9.21\times10^{-4}$ | $6.54\times10^{-4}$ |
| 35 | $6.66\times10^{-6}$ | $6.37\times10^{-6}$ | $1.65\times10^{-6}$ | $8.41\times10^{-6}$ | $6.03\times10^{-3}$ | $4.63\times10^{-6}$ | $1.06\times10^{-4}$ | $8.79\times10^{-5}$ | $2.29\times10^{-3}$ | $9.58\times10^{-4}$ |
| 36 | $1.91\times10^{-5}$ | $7.00\times10^{-6}$ | $1.20\times10^{-6}$ | $1.43\times10^{-5}$ | $2.96\times10^{-3}$ | $1.40\times10^{-5}$ | $2.12\times10^{-4}$ | $7.78\times10^{-5}$ | $2.91\times10^{-3}$ | $1.35\times10^{-3}$ |
| 37 | $1.97\times10^{-5}$ | $3.28\times10^{-6}$ | $1.45\times10^{-6}$ | $6.68\times10^{-6}$ | $4.66\times10^{-3}$ | $2.25\times10^{-6}$ | $8.96\times10^{-5}$ | $6.45\times10^{-5}$ | $5.15\times10^{-4}$ | $1.64\times10^{-4}$ |
| 38 | $7.09\times10^{-6}$ | $9.87\times10^{-6}$ | $6.87\times10^{-7}$ | $2.35\times10^{-6}$ | $5.18\times10^{-3}$ | $6.20\times10^{-5}$ | $2.60\times10^{-4}$ | $1.49\times10^{-4}$ | $1.85\times10^{-2}$ | $2.20\times10^{-4}$ |

Table S8. Cancer risk (CR) estimate of As and Pb in children (20Kg) and adults (70Kg).

| Sample numbers | As | | | | Pb | | | |
|---|---|---|---|---|---|---|---|---|
| | Children | | Adults | | Children | | Adults | |
| | He | Am | He | Am | He | Am | He | Am |
| 1 | $3.03\times10^{-7}$ | $1.11\times10^{-8}$ | $1.42\times10^{-7}$ | $4.13\times10^{-8}$ | $2.07\times10^{-9}$ | $2.40\times10^{-10}$ | $9.69\times10^{-10}$ | 8.92×10-10 |
| 2 | $6.48\times10^{-7}$ | $1.40\times10^{-8}$ | $3.03\times10^{-7}$ | $5.22\times10^{-8}$ | $6.41\times10^{-9}$ | $1.98\times10^{-10}$ | $3.00\times10^{-9}$ | 7.34×10-10 |
| 3 | $5.49\times10^{-8}$ | $1.46\times10^{-8}$ | $2.57\times10^{-8}$ | $5.41\times10^{-8}$ | $2.77\times10^{-9}$ | $5.79\times10^{-10}$ | $1.30\times10^{-9}$ | 2.15×10-9 |
| 4 | $1.62\times10^{-7}$ | $7.82\times10^{-9}$ | $7.56\times10^{-8}$ | $2.91\times10^{-8}$ | $4.85\times10^{-9}$ | $1.74\times10^{-10}$ | $2.27\times10^{-9}$ | 6.46×10-10 |
| 5 | $8.79\times10^{-8}$ | $1.28\times10^{-8}$ | $4.11\times10^{-8}$ | $4.76\times10^{-8}$ | $1.37\times10^{-9}$ | $3.95\times10^{-10}$ | $6.40\times10^{-10}$ | $1.47\times10^{-9}$ |
| 6 | $8.60\times10^{-8}$ | $1.09\times10^{-8}$ | $4.02\times10^{-8}$ | $4.07\times10^{-8}$ | $3.00\times10^{-9}$ | $3.00\times10^{-10}$ | $1.40\times10^{-9}$ | $1.11\times10^{-9}$ |
| 7 | $8.98\times10^{-8}$ | $8.97\times10^{-9}$ | $4.21\times10^{-8}$ | $3.33\times10^{-8}$ | $3.68\times10^{-9}$ | $3.61\times10^{-10}$ | $1.72\times10^{-9}$ | $1.34\times10^{-9}$ |
| 8 | $1.08\times10^{-7}$ | $8.57\times10^{-9}$ | $5.07\times10^{-8}$ | $3.18\times10^{-8}$ | $4.80\times10^{-9}$ | $4.31\times10^{-10}$ | $2.25\times10^{-9}$ | $1.60\times10^{-9}$ |
| 9 | $1.68\times10^{-7}$ | $1.86\times10^{-8}$ | $7.85\times10^{-8}$ | $6.92\times10^{-8}$ | $4.43\times10^{-9}$ | $5.91\times10^{-10}$ | $2.07\times10^{-9}$ | $2.20\times10^{-9}$ |
| 10 | $6.63\times10^{-8}$ | $1.13\times10^{-8}$ | $3.11\times10^{-8}$ | $4.21\times10^{-8}$ | $1.36\times10^{-9}$ | $3.48\times10^{-10}$ | $6.37\times10^{-10}$ | $1.29\times10^{-9}$ |
| 11 | $9.37\times10^{-8}$ | $8.00\times10^{-9}$ | $4.39\times10^{-8}$ | $2.97\times10^{-8}$ | $2.79\times10^{-9}$ | $3.66\times10^{-10}$ | $1.30\times10^{-9}$ | $1.36\times10^{-9}$ |
| 12 | $1.47\times10^{-7}$ | $8.23\times10^{-9}$ | $6.88\times10^{-8}$ | $3.06\times10^{-8}$ | $2.75\times10^{-9}$ | $2.16\times10^{-10}$ | $1.29\times10^{-9}$ | $8.04\times10^{-10}$ |
| 13 | $8.04\times10^{-8}$ | $9.00\times10^{-9}$ | $3.76\times10^{-8}$ | $3.34\times10^{-8}$ | $3.27\times10^{-9}$ | $3.97\times10^{-10}$ | $1.53\times10^{-9}$ | $1.47\times10^{-9}$ |
| 14 | $1.16\times10^{-7}$ | $1.50\times10^{-8}$ | $5.45\times10^{-8}$ | $5.56\times10^{-8}$ | $2.17\times10^{-9}$ | $3.48\times10^{-10}$ | $1.02\times10^{-9}$ | $1.29\times10^{-9}$ |
| 15 | $7.09\times10^{-8}$ | $1.16\times10^{-8}$ | $3.32\times10^{-8}$ | $4.31\times10^{-8}$ | $3.41\times10^{-9}$ | $7.17\times10^{-10}$ | $1.59\times10^{-9}$ | $2.66\times10^{-9}$ |
| 16 | $8.81\times10^{-8}$ | $1.13\times10^{-8}$ | $4.12\times10^{-8}$ | $4.19\times10^{-8}$ | $3.57\times10^{-9}$ | $2.95\times10^{-10}$ | $1.67\times10^{-9}$ | $1.10\times10^{-9}$ |
| 17 | $3.75\times10^{-7}$ | $8.47\times10^{-9}$ | $1.76\times10^{-7}$ | $3.15\times10^{-8}$ | $4.22\times10^{-9}$ | $5.28\times10^{-10}$ | $1.98\times10^{-9}$ | $1.96\times10^{-9}$ |
| 18 | $2.80\times10^{-7}$ | $1.15\times10^{-8}$ | $1.31\times10^{-7}$ | $4.27\times10^{-8}$ | $3.75\times10^{-9}$ | $5.13\times10^{-10}$ | $1.76\times10^{-9}$ | $1.91\times10^{-9}$ |
| 19 | $2.16\times10^{-7}$ | $1.31\times10^{-8}$ | $1.01\times10^{-7}$ | $4.86\times10^{-8}$ | $9.61\times10^{-9}$ | $1.01\times10^{-9}$ | $4.50\times10^{-9}$ | $3.74\times10^{-9}$ |
| 20 | $5.79\times10^{-7}$ | $1.00\times10^{-8}$ | $2.71\times10^{-7}$ | $3.72\times10^{-8}$ | $1.28\times10^{-8}$ | $5.18\times10^{-10}$ | $6.01\times10^{-9}$ | $1.92\times10^{-9}$ |
| 21 | $5.19\times10^{-8}$ | $4.59\times10^{-9}$ | $2.43\times10^{-8}$ | $1.70\times10^{-8}$ | $4.39\times10^{-9}$ | $5.25\times10^{-10}$ | $2.06\times10^{-9}$ | $1.95\times10^{-9}$ |
| 22 | $1.73\times10^{-7}$ | $1.35\times10^{-8}$ | $8.11\times10^{-8}$ | $5.02\times10^{-8}$ | $4.72\times10^{-9}$ | $3.97\times10^{-10}$ | $2.21\times10^{-9}$ | $1.47\times10^{-9}$ |
| 23 | $1.45\times10^{-7}$ | $1.05\times10^{-8}$ | $6.80\times10^{-8}$ | $3.90\times10^{-8}$ | $4.82\times10^{-9}$ | $3.15\times10^{-10}$ | $2.25\times10^{-9}$ | $1.17\times10^{-9}$ |
| 24 | $4.29\times10^{-7}$ | $8.01\times10^{-9}$ | $2.01\times10^{-7}$ | $2.98\times10^{-8}$ | $6.82\times10^{-9}$ | $2.86\times10^{-10}$ | $3.19\times10^{-9}$ | $1.06\times10^{-9}$ |
| 25 | $1.14\times10^{-6}$ | $8.81\times10^{-9}$ | $5.32\times10^{-7}$ | $3.27\times10^{-8}$ | $2.62\times10^{-9}$ | $2.76\times10^{-10}$ | $1.23\times10^{-9}$ | $1.03\times10^{-9}$ |
| 26 | $1.39\times10^{-7}$ | $8.22\times10^{-9}$ | $6.48\times10^{-8}$ | $3.05\times10^{-8}$ | $3.46\times10^{-9}$ | $3.36\times10^{-10}$ | $1.62\times10^{-9}$ | $1.25\times10^{-9}$ |
| 27 | $1.31\times10^{-7}$ | $1.05\times10^{-8}$ | $6.13\times10^{-8}$ | $3.89\times10^{-8}$ | $4.72\times10^{-9}$ | $5.64\times10^{-10}$ | $2.21\times10^{-9}$ | $2.10\times10^{-9}$ |
| 28 | $1.25\times10^{-7}$ | $5.70\times10^{-9}$ | $5.83\times10^{-8}$ | $2.12\times10^{-8}$ | $9.17\times10^{-9}$ | $5.96\times10^{-10}$ | $4.29\times10^{-9}$ | $2.22\times10^{-9}$ |
| 29 | $8.04\times10^{-8}$ | $1.28\times10^{-8}$ | $3.76\times10^{-8}$ | $4.77\times10^{-8}$ | $2.77\times10^{-9}$ | $3.54\times10^{-10}$ | $1.29\times10^{-9}$ | $1.32\times10^{-9}$ |
| 30 | $8.75\times10^{-8}$ | $1.00\times10^{-8}$ | $4.09\times10^{-8}$ | $3.73\times10^{-8}$ | $3.25\times10^{-9}$ | $7.33\times10^{-10}$ | $1.52\times10^{-9}$ | $2.72\times10^{-9}$ |
| 31 | $3.36\times10^{-7}$ | $1.37\times10^{-8}$ | $1.57\times10^{-7}$ | $5.09\times10^{-8}$ | $4.12\times10^{-9}$ | $5.61\times10^{-10}$ | $1.93\times10^{-9}$ | $2.08\times10^{-9}$ |
| 32 | $2.02\times10^{-7}$ | $7.92\times10^{-9}$ | $9.46\times10^{-8}$ | $2.94\times10^{-8}$ | $2.30\times10^{-9}$ | $3.20\times10^{-10}$ | $1.08\times10^{-9}$ | $1.19\times10^{-9}$ |
| 33 | $1.83\times10^{-7}$ | $1.63\times10^{-8}$ | $8.57\times10^{-8}$ | $6.06\times10^{-8}$ | $2.31\times10^{-9}$ | $2.13\times10^{-10}$ | $1.08\times10^{-9}$ | $7.91\times10^{-10}$ |
| 34 | $2.14\times10^{-7}$ | $1.37\times10^{-8}$ | $1.00\times10^{-7}$ | $5.08\times10^{-8}$ | $8.72\times10^{-9}$ | $4.77\times10^{-10}$ | $4.08\times10^{-9}$ | $1.77\times10^{-9}$ |
| 35 | $1.02\times10^{-7}$ | $1.06\times10^{-8}$ | $4.77\times10^{-8}$ | $3.95\times10^{-8}$ | $2.42\times10^{-9}$ | $2.91\times10^{-10}$ | $1.13\times10^{-9}$ | $1.08\times10^{-9}$ |
| 36 | $2.04\times10^{-7}$ | $9.42\times10^{-9}$ | $9.56\times10^{-8}$ | $3.50\times10^{-8}$ | $6.92\times10^{-9}$ | $3.20\times10^{-10}$ | $3.24\times10^{-9}$ | $1.19\times10^{-9}$ |
| 37 | $8.62\times10^{-8}$ | $7.81\times10^{-9}$ | $4.03\times10^{-8}$ | $2.90\times10^{-8}$ | $7.15\times10^{-9}$ | $1.50\times10^{-10}$ | $3.35\times10^{-9}$ | $5.57\times10^{-10}$ |
| 38 | $2.50\times10^{-7}$ | $1.81\times10^{-8}$ | $1.17\times10^{-7}$ | $6.71\times10^{-8}$ | $2.58\times10^{-9}$ | $4.52\times10^{-10}$ | $1.21\times10^{-9}$ | $1.68\times10^{-9}$ |

Table S9. Correlation coefficients among four toxic elements in the same tissues collected from red swamp crayfish (*Procambarus clarkii*).

| | Gi | | | | He | | | | Am | | | | Hc | | | |
|---|---|---|---|---|---|---|---|---|---|---|---|---|---|---|---|---|
| | **As** | **Pb** | **Cd** | **Cu** | **As** | **Pb** | **Cd** | **Cu** | **As** | **Pb** | **Cd** | **Cu** | **Hg** | **As** | **Pb** | **Cd** |
| **Hg** | 0.012 | 0.002 | 0.175 | 0.145 | **-.400**$^{*}$ | -.132 | 0.181 | -.040 | -.144 | -.116 | -.130 | 0.026 | 1 | 0.149 | -.125 | 0.078 |
| **As** | 1 | **0.616**$^{**}$ | 0.074 | 0.040 | 1 | 0.236 | -.068 | -.109 | 1 | 0.164 | 0.303 | 0.113 | | 1 | 0.136 | 0.179 |
| **Pb** | | 1 | **0.359**$^{*}$ | 0.254 | | 1 | -.153 | -.021 | | 1 | 0.057 | 0.208 | | | 1 | 0.096 |
| **Cd** | | | 1 | **0.405**$^{*}$ | | | 1 | 0.202 | | | 1 | -.073 | | | | |

Hc, Head capsule; Gi,Gill; He,Hepatopancreas; Am,Abdominal muscle; "*" indicates $P < 0.05$, "**" indicates $P < 0.01$.
